# Phage‐displayed heptapeptide sequence conjugation significantly improves the specific targeting ability of antimicrobial peptides against *Staphylococcus aureus*

**DOI:** 10.1002/mlf2.12123

**Published:** 2024-05-27

**Authors:** Tao Wang, Peng Tan, Qi Tang, Chenlong Zhou, Yakun Ding, Shenrui Xu, Mengda Song, Huiyang Fu, Yucheng Zhang, Xiaohui Zhang, Yueyu Bai, Zhihong Sun, Xi Ma

**Affiliations:** ^1^ State Key Laboratory of Animal Nutrition and Feeding, College of Animal Science and Technology China Agricultural University Beijing China; ^2^ Luoyang Key Laboratory of Animal Genetic and Breeding, College of Animal Science Henan University of Science and Technology Luoyang China; ^3^ Key Laboratory of Innovative Utilization of Indigenous Cattle and Sheep Germplasm Resources (Co‐construction by Ministry and Province), Ministry of Agriculture and Rural Affairs Zhengzhou University Zhengzhou China; ^4^ Animal Health Supervision in Henan Province Zhengzhou China; ^5^ Laboratory for Bio‐Feed and Molecular Nutrition, Department of Animal Science and Technology Southwest University Chongqing China

**Keywords:** antimicrobial peptides, membrane disruption mechanism, microbial infection, phage display technology, *Staphylococcus aureus*

## Abstract

Broad‐spectrum antibacterial drugs often lack specificity, leading to indiscriminate bactericidal activity, which can disrupt the normal microbial balance of the host flora and cause unnecessary cytotoxicity during systemic administration. In this study, we constructed a specifically targeted antimicrobial peptide against *Staphylococcus aureus* by introducing a phage‐displayed peptide onto a broad‐spectrum antimicrobial peptide and explored its structure–function relationship through one‐factor modification. SFK2 obtained by screening based on the selectivity index and the targeting index showed specific killing ability against *S. aureus*. Moreover, SFK2 showed excellent biocompatibility in mice and piglet, and demonstrated significant therapeutic efficacy against *S. aureus* infection. In conclusion, our screening of phage‐derived heptapeptides effectively enhances the specific bactericidal ability of the antimicrobial peptides against *S. aureus*, providing a theoretical basis for developing targeted antimicrobial peptides.

## Impact statement

In treating diseases caused by microbial infections, broad‐spectrum antimicrobial peptides often lead to dysbiosis of the host flora and are prone to unwanted toxicity. In this study, we used phage display technology to screen for heptapeptide sequences that can specifically bind *Staphylococcus aureus* and modify them to obtain antimicrobial peptides with specific recognition abilities. The targeted peptide, SFK2, exerted antimicrobial effects by disturbing the structure of the bacterial membrane and showed good therapeutic effects in vivo. This study provides a reference for designing and developing targeted antimicrobial peptides.

## INTRODUCTION

Antibiotics have been widely used in clinical treatments to effectively combat microbial infections. Bacterial resistance to antibiotics occurs naturally. However, extensive and irregular use of antibiotics has accelerated the evolution and mutation of drug‐resistant strains^1^, ^2^, ^3^, ^4^, ^5^. Therefore, it is important to identify and develop new antibacterial drugs that are safe and efficient. Antimicrobial peptides have good application prospects because they are less likely to cause drug resistance and are expected to become substitutes for antibiotics^6^, ^7^, ^8^.

Microorganisms play a very important role in the host gut ecosystem^9^, ^10^. However, most antimicrobial peptides with broad‐spectrum antimicrobial activity are prone to cause an imbalance in the host intestinal flora in response to pathogenic bacterial infections, thereby disrupting the dynamic equilibrium between healthy flora and the immune system^11^, ^12^, ^13^. In addition, if a local infection is treated with systemic or high‐concentration broad‐spectrum antimicrobial peptides, unnecessary toxicity caused by off‐target effects will inevitably occur, which will affect the therapeutic effect^14^.

Phage display is a molecular technology based on phage DNA modifications. A phage peptide library was constructed by introducing a DNA sequence encoding a foreign protein at a specific position on a nucleotide sequence encoding a phage surface protein^15^. Phage‐displayed peptides have the advantages of strong specificity for target molecules, low operational cost, high purity, and easy mass production^16^. Therefore, they are widely used in screening targeted drugs, preparing vaccines, preparing monoclonal antibodies, and tumor detection and diagnosis^17^, ^18^, ^19^, ^20^.

**Figure 1:**
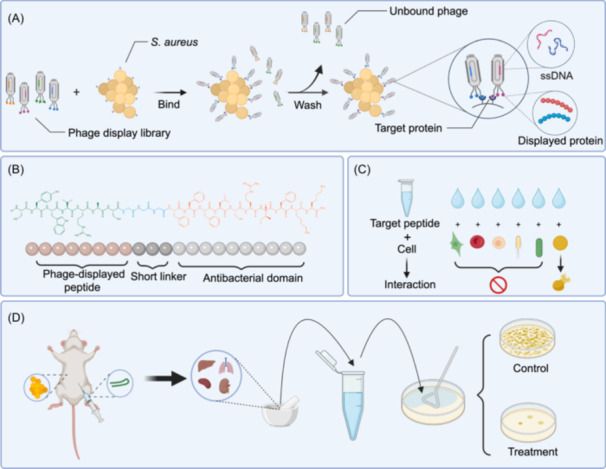
Schematic diagram of phage display technology, the chemical structure of the peptide, specific interaction of the target peptide, and in vivo antibacterial activity. (A) Phage display technology. (B) Chemical structure of peptide. (C) Specific interaction of target peptide. (D) In vivo antibacterial activity.

This study designed chimeric peptides based on the phage display‐screened heptapeptide sequence (SYWVRAS) and the broad‐spectrum antimicrobial peptide Temporin‐SHf (FFFLSRIF). The design concept mainly consisted of four parts: (I) The heptapeptide sequence that can bind to *Staphylococcus aureus* was screened from the peptide library using phage display technology. The heptapeptide sequence corresponding to the phage clone with the strongest specific binding ability was screened using enzyme‐linked immunosorbent assay (ELISA) as the targeting domain. (II) The broad‐spectrum antimicrobial peptide Temporin‐SHf, which is rich in hydrophobic amino acid residues in the antimicrobial peptide database, was selected as the antibacterial domain, and provided certain hydrophobicity for the chimeric peptide. (III) Three glycine (GGG) residues were used as a linker to connect the heptapeptide sequence and the antibacterial domain. (IV) Adding cationic amino acids (R or K) to the C‐terminus of the peptide increased the positive charge of the chimeric peptide and improved its antibacterial activity. We measured the minimum inhibitory concentration (MIC), bactericidal kinetics, hemolytic activity, and cytotoxicity of the candidate peptides in vitro. We selected SFK2, which had the strongest targeting ability, as the target peptide and studied its antibacterial mechanism and targeting ability using the lipoteichoic acid (LTA) binding test, the cell membrane depolarization test, electron microscopy, and flow cytometry. Finally, we explored the biological safety of the peptide and evaluated its therapeutic effect in mice and piglet, providing a reference for the further development of antimicrobial peptides with specific targeting ability (Figure 1).

## RESULTS

### Designing peptides based on phage display technology

*S. aureus* ATCC 6538 was used as the target bacterium, and three rounds of subtractive screening were performed using the phage display technique (Figure 1). *S. aureus* was immobilized in 96‐well plates and blocked with blocking buffer. Target molecules were incubated with the phage peptide library at room temperature. Phages with weak binding ability to *S. aureus* ATCC 6538 were discarded during Tris buffered saline + 0.1% [v/v] Tween‐20 (TBST) washing. Phages bound to *S. aureus* ATCC 6538 were eluted and used in the next round of screening. As the number of elutions increased, phages with strong binding ability to *S. aureus* ATCC 6538 were gradually enriched (Table S1).

Twenty‐four blue plaques were randomly selected from the titer plate using a pipette and amplified using a diluted *Escherichia coli* (*E. coli*) 2738 culture. DNA from the 24 phage clones was extracted using an ethanol precipitation method. The extracted phage DNA was sequenced by Bioengineering Co. The results of the six‐phage DNA sequencing showed overlapping peaks. The DNA sequencing results of the remaining 18 phages were analyzed. The enzymatic cut site of *Eag*I (CGGCCG), followed by bases 11–32, was the heptapeptide insertion sequence, and the DNA sequences were translated according to the codon table translation (Table S2). The results are shown in Table S3. The highest percentage of the heptapeptide sequence MHHRHTQ was found in the phage clone.

Because artifacts inevitably appear during the panning process, it was necessary to determine the affinity of the selected phage clones for *S. aureus* using ELISA. The results showed that phage clone #17 had the highest absorbance at 450 nm, indicating that the heptapeptide sequence SYWVRAS had the strongest affinity for *S. aureus* ATCC 6538 and was a positive phage clone (Figure S1). Therefore, we speculate that the main component of the enzyme plate is polystyrene and that peptides rich in aromatic residues (F, Y, W) and H selectively bind to polystyrene when the elution test is performed on an enzyme plate coated with target molecules^21^.

The heptapeptide sequence, SYWVRAS, was identified as the target structural domain by phage display screening. According to the literature, the charge number and hydrophobic amino acid percentage of peptides possessing antimicrobial activity are controlled at 6–8 and 40%–60%, respectively^22^. Therefore, we selected a natural antimicrobial peptide, Temporin‐SHf (amino acid series FFFLSRIF), with a high hydrophobic amino acid percentage and broad‐spectrum antimicrobial activity from the antimicrobial peptide database (https://aps.unmc.edu/AP/) as the antimicrobial domain^23^. The targeting and antimicrobial domains were linked by GGG as a linker sequence so that the two structural domains remained structurally and functionally independent. Subsequently, a series of antimicrobial peptides were designed by adding cationic amino acids (R or K) to increase the positive charge number and amidation modification of the C‐terminus to further enhance the antimicrobial activity (Table S4).

### Antimicrobial activity, biocompatibility, salt stability, and secondary structure of peptides

The MICs of the antimicrobial peptides against *S. aureus*, *Staphylococcus epidermidis* (*S. epidermidis*), *E. coli*, and *Pseudomonas aeruginosa* (*P. areuginosa*) were determined using the microbroth dilution method. The results are shown in Tables 1 and 2: Due to the targeting domain (SYWVRAS), most of the designed antimicrobial peptides showed good antibacterial effect against *S. aureus* at low concentrations. In addition, the peptide solution at a concentration of 64 μM did not show an inhibitory effect against *E. coli* ATCC 25922, *P. aeruginosa* ATCC 27853, and *P. aeruginosa* ATCC 15442. As the number of positive charges increased, the antimicrobial peptide showed inhibitory activity against other nontarget bacteria. For example, in addition to its antibacterial activity against *S. aureus*, SFK3 has antibacterial effects on *E. coli* ATCC K88 and *E. coli* ATCC K99.

**Table 1:** Minimum inhibitory concentration (MIC) of peptide against Gram‐positive bacteria.

	MIC of Gram‐positive bacteria (μM)		
Peptides	*S. aureus* 1882	*S. aureus* 6538	*S. epidermidis* 12228
SF	2	16	>64
SFR	2	2	>64
SFK	2	4	>64
SFR2	1	4	>64
SFK2	2	2	>64
SFR3	4	16	>64
SFK3	1	2	>64
Temporin‐SHf	>64	>64	>64

*S. aureus*, *Staphylococcus aureus*; *S. epidermidis*, *Staphylococcus epidermidis*.

**Table 2:** MIC of peptide against Gram‐negative bacteria.

	MIC of Gram‐negative bacteria (μM)				
Peptide	*E. coli* 25922	*E. coli* K88	*E. coli* K99	*P. aeruginosa* 27853	*P. aeruginosa* 15442
SF	>64	>64	>64	>64	>64
SFR	>64	32	32	>64	>64
SFK	>64	32	64	>64	>64
SFR2	>64	32	64	>64	>64
SFK2	>64	>64	>64	>64	>64
SFR3	>64	32	>64	>64	>64
SFK3	>64	16	16	>64	>64
Temporin‐SHf	>64	>64	>64	>64	>64

*E. coli*, *Escherichia coli*; *P. aeruginosa*, *Pseudomonas aeruginosa*.

**Figure 2:**
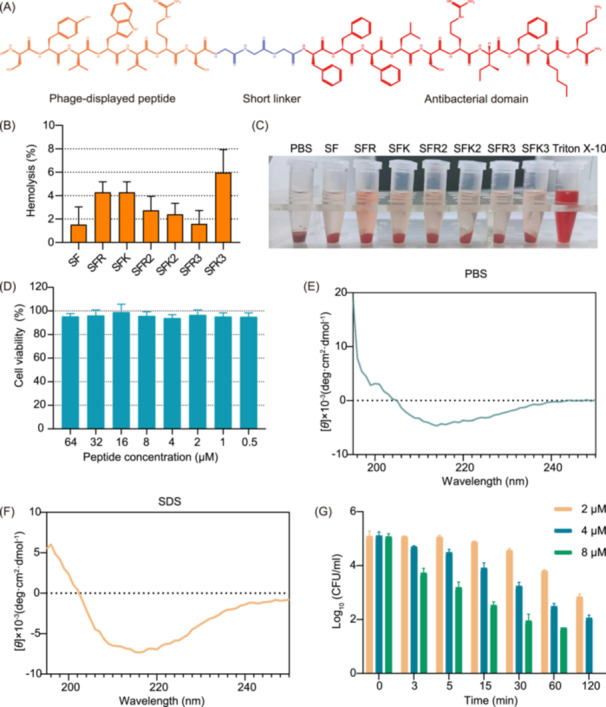
Structure and biocompatibility of peptides. (A) Chemical structure formula of SFK2. (B) Percentage hemolysis of erythrocytes after 2 h of treatment with different concentrations of designed peptides. (C) Pictures of designed peptides and Triton X‐100 after 2 h of incubation with erythrocytes. (D) Survival of HEK293T cells after 2 h of treatment with different concentrations of SFK2. (E, F) Determination of the secondary structure of SFK2 in 10 mM PBS (pH = 7.4) (E) and 30 mM SDS (F). (G) Killing kinetics of SFK2 against *Staphylococcus aureus* ATCC 6538.

Antimicrobial peptides need to possess excellent antimicrobial activity and biocompatibility in vivo. A series of parameters such as charge number, hydrophobicity, sequence length, and amphipathicity can affect the hemolytic activity of peptides against red blood cells. The minimal hemolytic concentration (MHC) refers to the minimum concentration that causes hemolysis of 10 % of red blood cells, and is often used to evaluate the hemolytic activity of the peptides. Except for SFK3, the percentage of hemolysis at 128 μM was below 5% for all peptides. Hemolysis of the erythrocytes occurred after incubation with Triton X‐100, with complete fragmentation of the erythrocytes and a bright red color of the reaction system (Figure 2B,C and Table S5).

The selectivity index and targeting indices of the designed peptides were determined. The greater the selectivity index (SI = MHC/the geometric mean (GM) of MIC value of peptides to bacteria) of the peptide, the better its selectivity for cells. The targeting index TI = GM (*S. aureus*)/GM (other bacteria). The smaller the targeting index of the peptide, the better its specific antibacterial activity against *S. aureus*. Among the designed peptides SFR, SFR2, and SFK2 had the highest cell selectivity indices, and SFK2 had the lowest targeting index among the seven peptides (Table S6), indicating that it had the most specific antibacterial ability against *S. aureus*. Therefore, SFK2 was used as the target peptide in subsequent experiments (Figure 2A).

Subsequently, we determined the cytotoxicity of the antimicrobial peptide SFK2 on HEK293T cells using the 3‐(4,5‐dimethylthiazol‐2‐yl)‐2,5‐diphenyltetrazolium bromide (MTT) assay. The peptide did not cause obvious cytotoxicity after incubation with cells for 2 h (Figure 2D). These results indicate that the peptide showed good biocompatibility in vitro. Since the antimicrobial peptides are positively charged, they could bind to the negatively charged components of microbial cell membrane surfaces, such as phosphopeptides, through electrostatic interactions. Mammalian cells are eukaryotic cells, and compared with prokaryotic cells, the negatively charged components are mainly concentrated on the inner side of the cell rather than on the cell membrane surface. Therefore, antimicrobial peptides have less ability to interact with mammalian cells and are less likely to produce cytotoxicity^24^.

The loss of antibacterial activity of antimicrobial peptides in the physiological environment in vivo is the main factor that limits its clinical application. We determined the antibacterial activity of SFK2 under various physiological salt environments. The results are shown in Table S7. The MIC of SFK2 against *S. aureus* increased twofold in the presence of Na^+^, K^+^, NH_4_^+^, Mg^2+^, and Fe^3+^, whereas the antimicrobial activity did not change in the presence of Zn^2+^. In the in vivo environment, various ions compete with antimicrobial peptide cations for binding sites in the lipid moiety of the bacterial membrane, rendering them less active and unable to act effectively^25^. In addition, Fe^3+^ can combine with negatively charged components on the surface of cell membranes to increase its rigidity^26^. Although the antibacterial activity of SFK2 was affected in a physiological salt environment, its salt tolerance remained within an acceptable range.

The secondary structure of the peptide molecules in sodium dodecyl sulfate (SDS) and phosphate‐buffered saline (PBS) environments were investigated using circular dichroism changes to explore the structure–function relationship (Figure 2E,F). SDS and PBS were used to simulate the anion environment of the phospholipid bilayer and the aqueous environment, respectively. In the hydrophobic environment of the phospholipid bilayer simulated by SDS, SFK2 showed a clear positive peak at 195–198 nm and a clear negative peak at 217–218 nm, indicating the formation of a β‐fold conformation. It was found that β‐sheet peptides have higher cell selectivity than α‐helical peptides under the same hydrophobicity and charge condition^27^. In contrast, SFK2 did not show any significant secondary structure in PBS. This is similar to the structure of many β‐sheet peptides in aqueous environments^28^, ^29^, ^30^.

The bactericidal effect of SFK2 against *S. aureus* ATCC 6538 was determined by measuring the bactericidal kinetics (Figure 2G). At 1 × MIC, although SFK2 reduced the colony count in solution by 2 log units within 120 min, it could not completely kill *S. aureus* in the solution. At 4 × MIC, SFK2 completely killed *S. aureus* within 120 min, indicating good bactericidal ability.

### Main antimicrobial mechanisms of peptides

**Figure 3:**
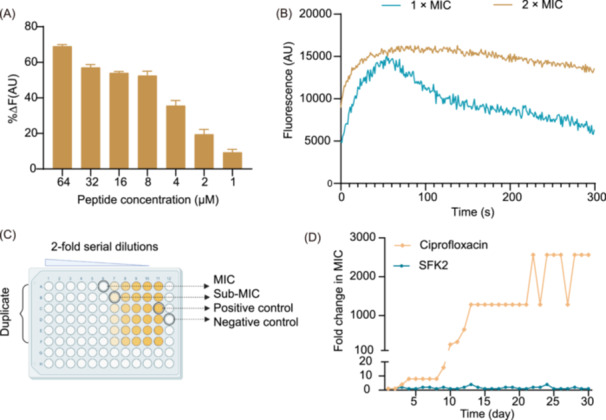
Antimicrobial mechanism of peptides and determination of drug resistance. (A) Binding capacity of different concentrations of SFK2 after 2 h incubation with lipoteichoic acid (LTA) of *Staphylococcus aureus*. (B) Cytoplasmic membrane permeability of *S. aureus* 6538 induced by SFK2. (C) Diagram of the drug resistance assay. Negative control, MHB medium without bacteria; postive control, MHB medium containing bacteria; Sub‐MIC, concentration of drug below the lowest inhibitory concentration. (D) Development of resistance to SFK2 and ciprofloxacin in *S. aureus* ATCC 6538 at Sub‐MIC concentrations.

LTA is a common negatively charged component found on the surface of *S. aureus* ^31^. We first measured the LTA binding affinity of SFK2 to *S. aureus* using the BODIPY‐TR‐cadaverine fluorescent dye substitution method to explore its targeting mechanism and antibacterial mechanisms. After co‐incubating the two for 1 h, the binding capacity of SFK2 to LTA showed a dose‐dependent effect (Figure 3A). At a concentration of 2 μM, SFK2 could bind 19.57% of LTA. At a concentration of 8 μM, SFK2 could bind 52.55% of LTA. Our design concept is based on cation and hydrophobicity, and based on the above experimental results, we inferred that the antimicrobial peptide exerts antibacterial activity by first binding to the LTA of *S. aureus*.

Lipids are an important component of bacterial cell membranes and a common binding site for antimicrobial drugs to exert their antimicrobial effects. A POPG/CL liposome suspension with a mass ratio 3:1 was used to simulate *S. aureus* membranes. We analyzed the interaction of fluorescein isothiocyanate (FITC)‐SFK2 with the liposomes using the Monolith NT.115 system. As shown in Figure S5, *F*_norm_ [‰] showed a typical S‐shaped binding curve with increased liposome concentration. The apparent dissociation constant *k*_d_ for both was derived as 166 µM by fitting to the liposome concentration.

Subsequently, the DiSC3(5) (3,3'‐Dipropylthiadicarbocyanine Iodide) fluorescent probe was used to detect the effects of SFK2 on the cytoplasmic membrane permeability of *S. aureus* ATCC 6538 (Figure 3B). SFK2‐induced fluorescence values at 1 × MIC concentration peaked at 54 s and then gradually quenched. The SFK2‐induced fluorescence value at 2 × MIC concentration peaked at 82 s, stabilized, and decreased slowly. Based on the above results, we speculate that SFK2 binds to LTA through electrostatic interactions and gathers on the surface of the cell membrane, destroying the plasma membrane to generate pores and ion channels, leading to its depolarization.

**Figure 4:**
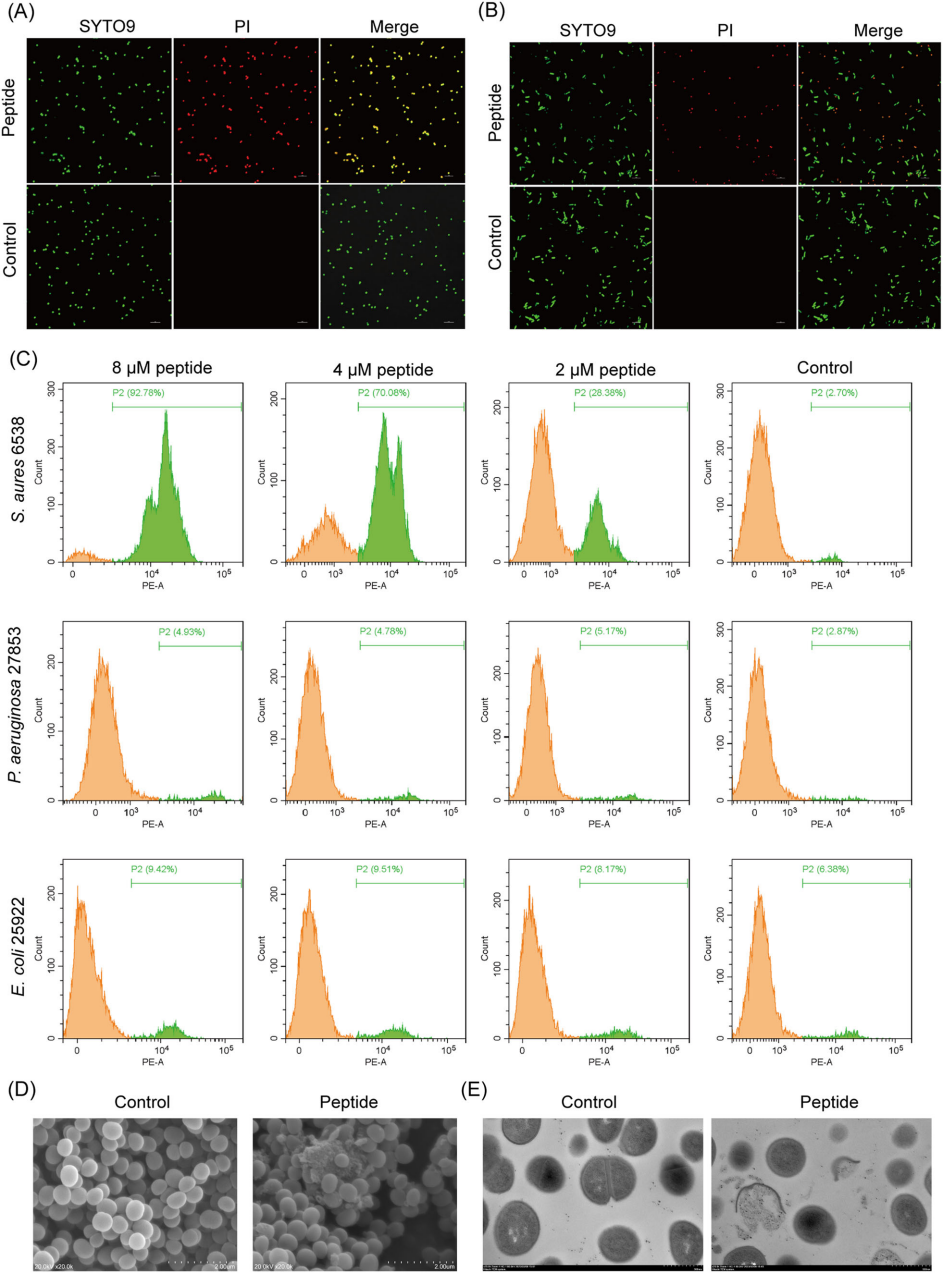
Effects of peptides on cell membrane integrity. (A) Laser confocal scanning microscopy images of *Staphylococcus aureus* ATCC 6538 after treatment with 2 μM SFK2 for 2 h. Scale bar: 5 μm. (B) Laser confocal scanning microscope images of *S. aureus* ATCC 6538 and *E. coli* ATCC 25922 after treatment with 2 μM SFK2 for 2 h. Scale bar: 5 μm. (C) Flow cytometry analysis of membrane integrity of *S. aureus* ATCC 6538, *Pseudomonas aeruginosa* ATCC 27853, and *E. coli* ATCC 25922 treated with different concentrations of SFK2. (D) SEM images of *S. aureus* ATCC 6538 treated with 2 μM SFK2 for 2 h. Scale bar of SEM: 2 μm. (E) TEM images of *S. aureus* ATCC 6538 treated with 2 μM SFK2 for 2 h. Scale bar of TEM: 500 nm. PI, Propidium Iodide; P2, proportion of cells generating PI fluorescent signals; SEM, scanning electron microscope; TEM, transmission electron microscopy.

To further verify the effect of SFK2 treatment on membrane integrity, *S. aureus* ATCC 6538 was incubated with a final concentration of 8 μM SFK2, followed by the addition of SYTO9 and propidium iodide (PI) fluorescent dyes, and the fluorescence distribution was observed under a laser scanning confocal microscope (Figure 4A). Green fluorescence (SYTO9) and red fluorescence (PI) of *S. aureus* ATCC 6538 induced under 8 μM of SFK2 overlapped, indicating severe disruption of its cell membrane and leading to cell death. Subsequently, to investigate the targeting ability of SFK2, *S. aureus* ATCC 6538 and *E. coli* ATCC 25922 were mixed 1:1, treated with 8 μM SFK2, and then SYTO9 and PI fluorescent dyes were added for observation using laser scanning confocal microscopy. The green fluorescence and red fluorescence of *S. aureus* ATCC 6538 were induced to overlap at 8 μM concentration of SFK2, while *E. coli* ATCC 25922 only emitted green fluorescence without red fluorescence (Figure 4B), indicating that its membrane integrity was not disrupted.

We aimed to demonstrate that *S. aureus* death is caused by SFK2 targeting. We incubated FITC‐labeled SFK2 with an *S. aureus* suspension for 2 h and observed it using laser confocal microscopy. As shown in Figure S6, green fluorescence overlapped with red fluorescence, and the peptides were uniformly distributed on the surface of each dead *S. aureus* membrane.

*S. aureus, P. aeruginosa*, and *E. coli* were incubated with different concentrations of SFK2, and their membrane integrity was quantified using flow cytometry. As shown in Figures 4C, 8, 4, and 2 μM of SFK2 induced 92.78%, 70.08%, and 28.38% of *S. aureus* mortality, respectively. However, *P. aeruginosa* and *E. coli* maintained more than 90% survival even at the final concentration of 8 μM SFK2, with negligible differences from the control. The above results indicate that SFK2 at a concentration of 8 μM induced a break in membrane integrity in more than 90% of *S. aureus* without affecting nontarget bacteria. It has a strong targeting ability.

To verify this hypothesis further, scanning electron microscope (SEM) and transmission electron microscopy (TEM) were used to observe the morphological changes in *S. aureus* treated with SFK2 (Figure 4). The surface of untreated *S. aureus* was intact and smoothly rounded under SEM (Figure 4D). *S. aureus* ruptured after 2 h of treatment with 2 μM SFK2, and there was an outflow of the contents outside the cell. Under the TEM (Figure 4E), the cell membrane structure of *S. aureus* in the control group was complete, and the cytoplasm was dense. After treatment with 2 μM SFK2 for 2 h, the cell membrane of *S. aureus* was ruptured and the cell contents were discharged.

The fold change in the MIC values of SFK2 and ciprofloxacin against *S. aureus* was determined using the continuous passaging method (Figure 3C,D). SFK2 showed no significant changes in MIC values during the 30‐day test, and no resistance problems were observed. In contrast, the MIC value of ciprofloxacin increased 16‐fold on Day 9 compared with the initial value. At the end of the trial, the MIC of ciprofloxacin against *S. aureus* was 2560 times that of the initial value. This finding is similar to those of the previous study^32^. The results of the mechanism of action study showed that the antibacterial activity of SFK2 relies mainly on the physical membrane‐breaking mechanism, and bacteria cannot change the membrane structure in a short period to resist peptide damage. In contrast, ciprofloxacin mainly acts on bacterial genetic material, inhibiting DNA synthesis and replication, leading to bacterial death, which can easily lead to drug resistance.

### In vivo biocompatibility evaluation of peptides

**Figure 5:**
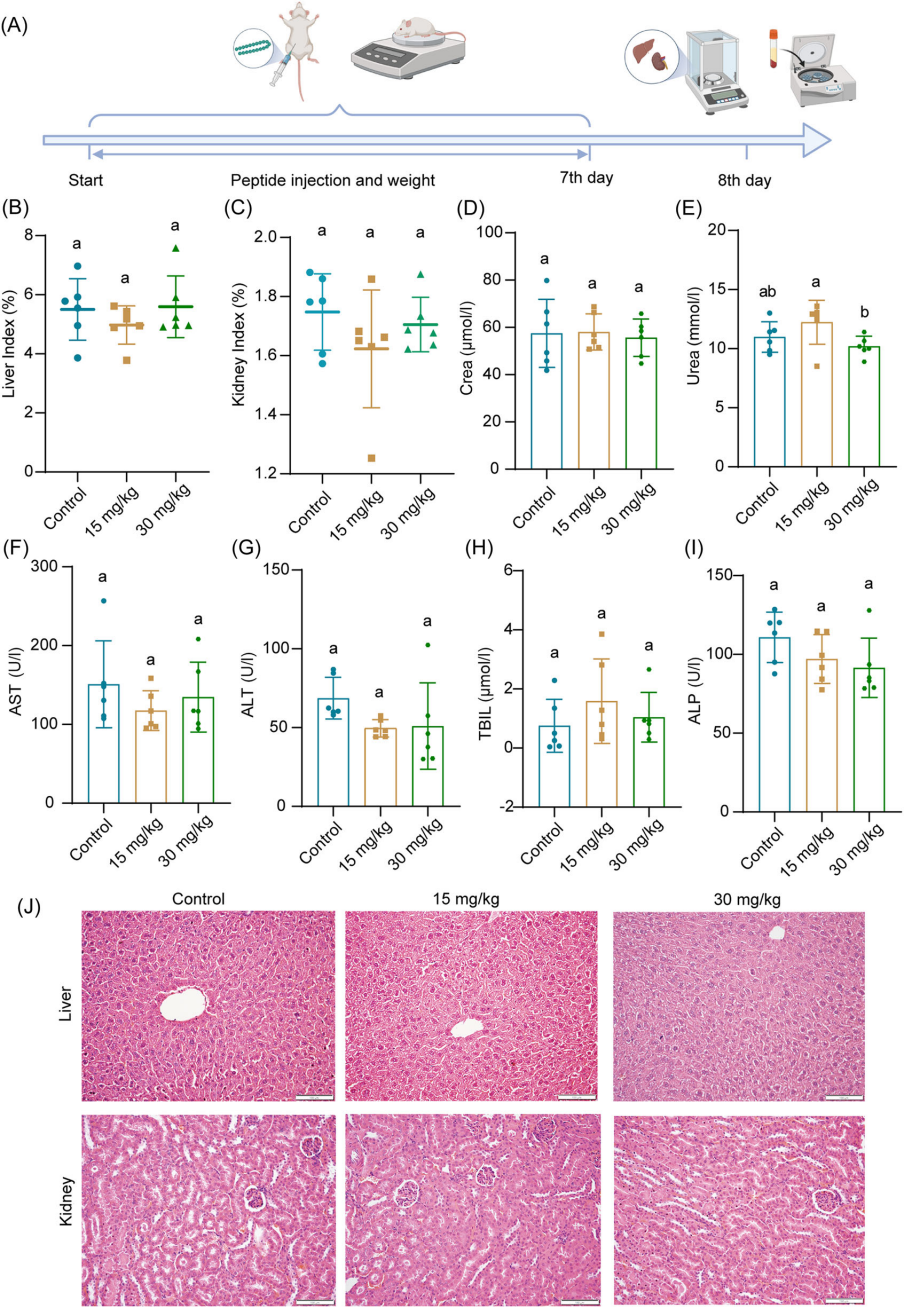
Biosafety evaluation of peptides in mice. (A) Schematic diagram of biocompatibility analysis of SFK2. (B,C) Change in the relative organ weights of liver (B) and kidney (C) in mice after 7 consecutive days of intraperitoneal injection of SFK2. (D–I) Changes in liver‐ and kidney‐related parameters in mice after 7 consecutive days of intraperitoneal injection of SFK2. Data are expressed as mean ± standard deviation, *n* = 6. (J) Histomorphological observation of the liver and kidney of mice after 7 consecutive days of intraperitoneal injection of different concentrations of SFK2. Scale bar: 100 μm. The same lowercase letter indicates that there is no significant difference (*p* > 0.05).The different lowercase letter indicates that there is significant difference (*p* < 0.05). ALT, alanine aminotransferase; ALP, alkaline phosphatase; AST, aspartate aminotransferase; Crea, creatinine; TBIL, total bilirubin.

In vivo animal testing are often used to evaluate the practical application of antimicrobial peptides. We assessed the biosafety of peptide‐based antibacterial drugs in vivo before conducting in vivo therapeutic trials. In this experiment, SFK2 was injected intraperitoneally at 15 and 30 mg/kg into mice for 7 consecutive days. The potential risk of SFK2 in mice was assessed using body weight changes, liver and kidney indices, liver‐ and kidney function‐related indices, and hematoxylin and eosin (H&E)‐stained histomorphological observations (Figure 5). The mice reverted to normal behavior 2 h after systemic administration. During the test period, there was no statistically significant difference in the body weights of mice in the low‐ and high‐dose groups compared with the control group (Figure S7). This peptide is metabolized in animals, mainly through the liver and kidneys. Therefore, the toxic side effects of SFK2 in the liver and kidneys should not be neglected. The relative organ weights of the liver and kidney, as well as liver‐ and kidney function‐related indices (creatinine, urea, aspartate aminotransferase, glutamate aminotransferase, total bilirubin, and serum alkaline phosphatase), were analyzed in mice (Figure 5B–I). There were no significant differences in the relative organ indices or related indices of the liver and kidneys between the groups of mice (except 15 mg/kg group for urea). This indicated that SFK2 did not cause damage to the liver or kidneys. Histological examination also revealed no significant abnormalities in the two peptide‐treated groups (Figure 5J). In addition, we evaluated the biosafety of SFK2 via a tail vein injection. The results showed that SFK2 had negligible toxic side effects in the mice (Figure S8).

### In vivo effect evaluation of peptide

**Figure 6:**
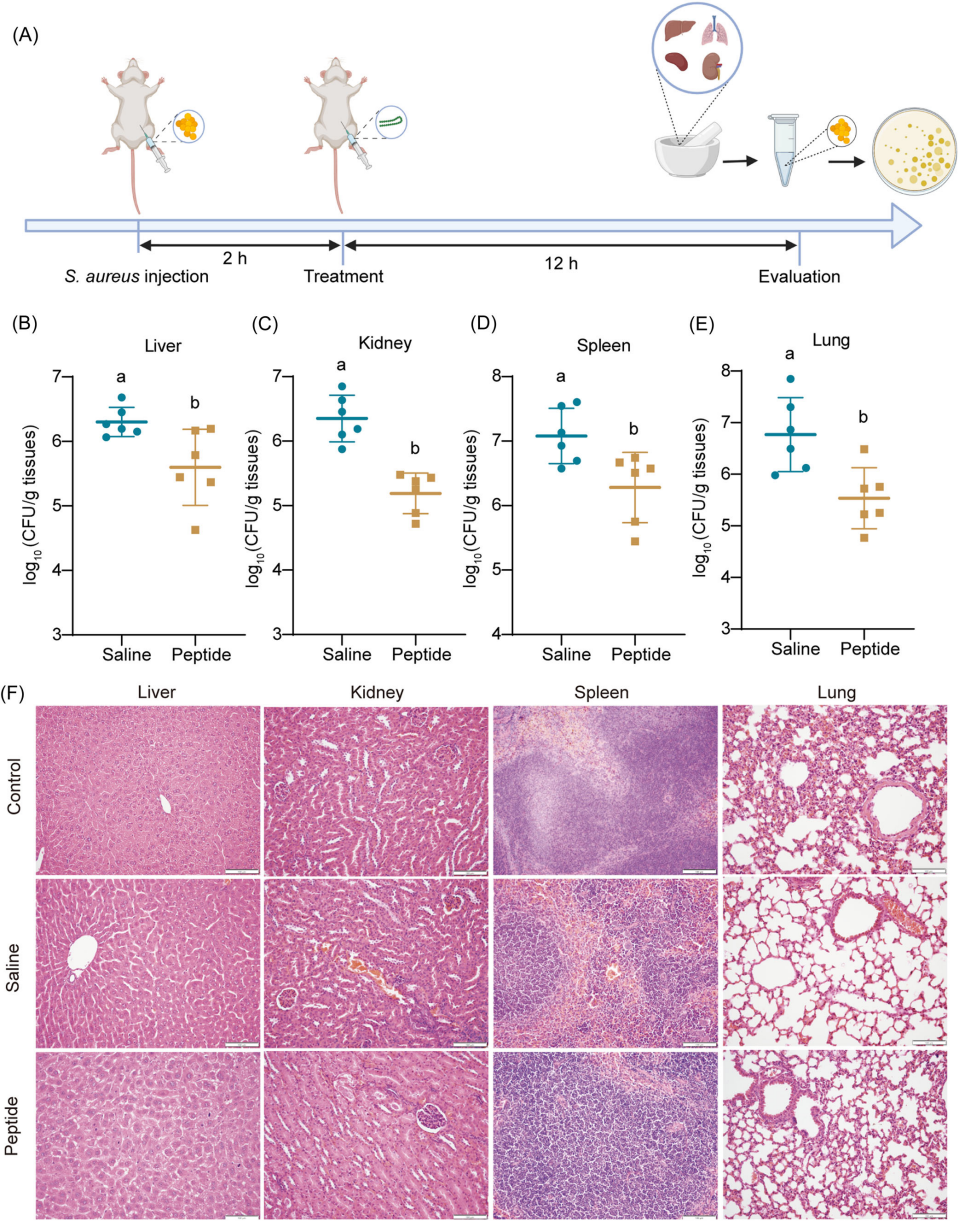
Evaluation of the therapeutic effect of peptides in mice in vivo. (A) Schematic representation of the therapeutic effect of SFK2 in mice. (B–E) Bacteria load in the liver, kidney, spleen, and lung of mice treated with saline and SFK2. Data are described as mean ± standard deviation, *n* = 6. The different lowercase letters indicates that there is significant difference (*p* < 0.05). (F) Histopathological sections of the liver, kidney, spleen, and lung of mice in the healthy group (control), the saline group (saline), and the SFK2 treatment group (peptide). Scale bar: 100 μm.

To evaluate the therapeutic effect of the antimicrobial peptide in vivo, a mouse bacteremia model was established by an intraperitoneal injection of 400 μl of *S. aureus* ATCC 6538 bacterial suspension (OD_600_ = 1.3). Two hours later, mice in the treatment group were injected intraperitoneally with 15 mg/kg SFK2. The saline‐treated group served as a control. After 12 h of treatment, the mice were euthanized, and tissues were collected and analyzed (Figure 6A). The bacterial load in the livers, kidneys, spleens, and lungs of SFK2‐treated mice was significantly lower than that in the saline‐treated group (Figure 6B–E). Hepatocellular damage, inflammatory cell infiltration, organ bleeding, and lung tissue breakdown in *S. aureus*‐infected mice were compared with those in healthy mice. Tissue damage was reduced after SFK2 treatment (Figure 6F). In addition, tail vein injection of SFK2 showed good therapeutic effects in the mouse infection model (Figure S9).

**Figure 7:**
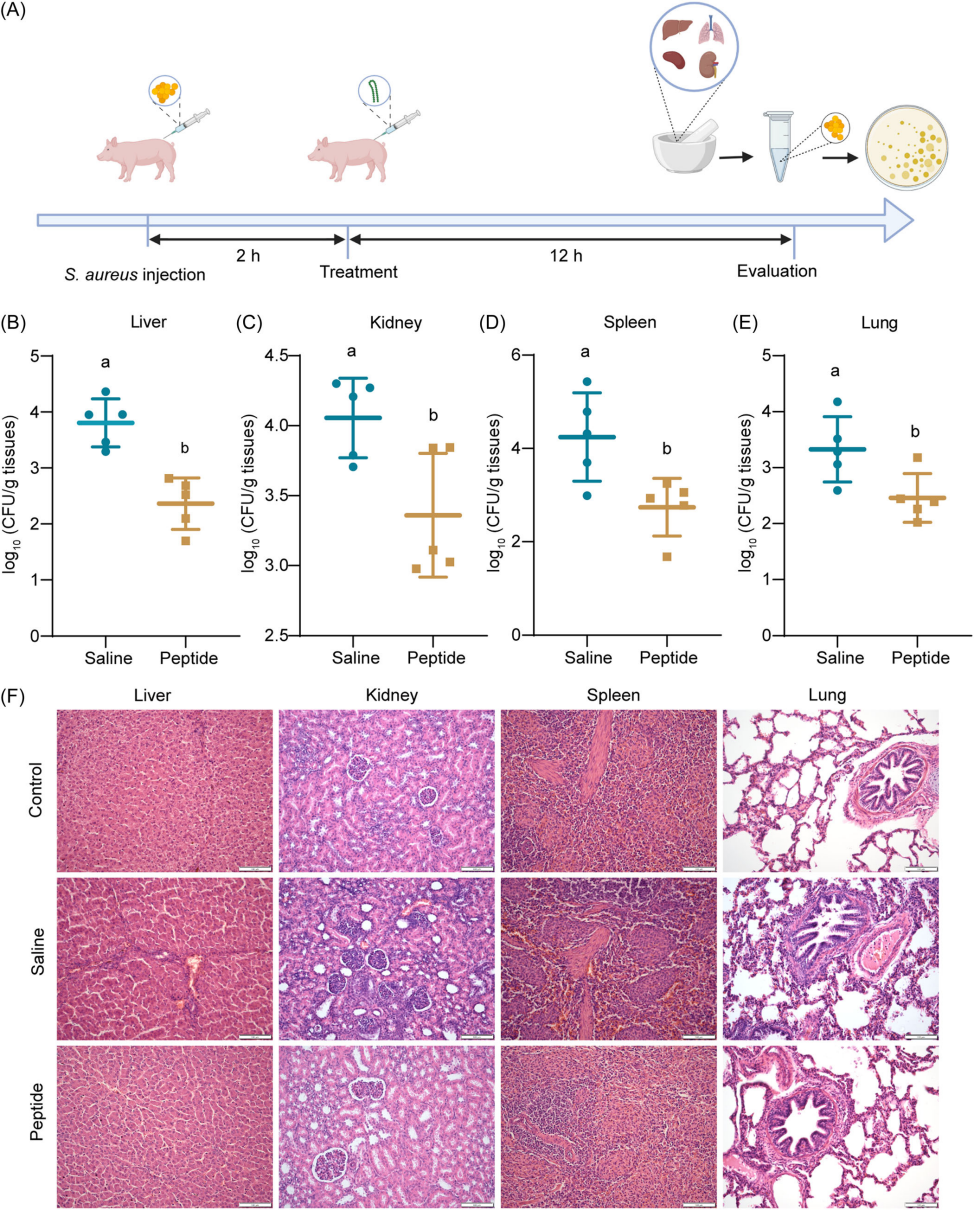
Evaluation of the therapeutic efficacy of peptides in piglets. (A) Schematic representation of the therapeutic effect of SFK2 in piglets. (B–E) Bacteria load in the liver, kidney, spleen, and lung of piglets treated with saline and SFK2 (3 mg/kg). Data are described as mean ± standard deviation, *n* = 5. The different lowercase letters indicates that there is significant difference (*p* < 0.05). (F) Histopathological sections of the liver, kidney, spleen, and lung of piglets in the healthy group (control), the saline group (saline), and the SFK2 treatment group (peptide). Scale bar: 100 μm.

We further explored the in vivo antimicrobial effect of SFK2 using piglets as target animals (Figure 7A). As shown in Figure 7, the bacterial loads in the livers, kidneys, spleens, and lungs were significantly reduced in SFK2‐treated piglets compared with those in the saline‐treated group (Figure 7B–E). Histological staining showed that organ inflammatory infiltration, tissue hemorrhage, and rupture symptoms were significantly reduced in piglets in the treatment group compared with those in the saline group (Figure 7F).

## DISCUSSION

Antibiotics are widely used in clinical treatment and animal production as an effective means to combat microbial infections. Antimicrobial peptides, as potential alternatives to antibiotics, have shown promising applications. However, owing to their broad‐spectrum antimicrobial activity, most antimicrobial peptides tend to cause imbalance in the host's intestinal flora. In addition, the systemic use of broad‐spectrum drugs for the treatment of localized infections may cause toxicity, which should not be overlooked.

Phage display technology involves the fusion of DNA fragments encoding exogenous proteins or small‐molecule polypeptides with phage genes encoding surface proteins, which then exist on the surface of phages by means of fusion proteins. Exogenous proteins and small‐molecule polypeptides specifically recognize and bind to target molecules. Therefore, this technology is widely used to screen targeted drugs, prepare vaccines and monoclonal antibodies, and monitor and diagnose tumors^17^. Researchers used the Ph.D.^TM^‐12 phage display peptide library to screen the affinity of the plague F1 antigen and then selenium was covalently bound to the high‐affinity phage and its corresponding peptide. The test results showed that both selenium‐containing phages and peptides maintained binding specificity to the plague F1 antigen and could specifically kill the target bacteria without affecting other bacteria^33^.

This study screened nine heptapeptide sequences from a random peptide library using phage display technology, with *S. aureus* 6538 as the target bacterium. The heptapeptide sequence with the highest affinity (SYWVRAS) was selected as the target domain. We further improved its antimicrobial activity by adding several cationic amino acids (R or K) at the C‐terminus after linking the heptapeptide sequence to Temporin‐SHf, which is rich in hydrophobic amino acids, via GGG as a linker. Compared with Temporin‐SHf, the designed peptide had higher specific antimicrobial activity against *S. aureus*. This suggests that the screened heptapeptide sequence effectively improves the specific antimicrobial activity after concatenation with the template peptide.

MLamP1 and MLamP6 are broad‐spectrum antimicrobial peptides with no antibacterial activity against *Clostridium perfringens*. Researchers have altered the antimicrobial spectrum of MLamP1 or MLamp6 by attaching the dodecapeptide sequence F6 to MLamP1 or MLamp6 using GGG^34^. The hybrid peptides F6P6 and P6F6 showed antibacterial activity against *C. perfringens* but did not kill other bacteria. Zhang et al. developed an antimicrobial peptide against a specific septic pathogen based on phage display technology, suggesting that it is feasible to design specifically targeted sepsis^35^. The specifically targeted antimicrobial peptide VTK‐LL37 effectively killed pathogenic bacteria and inhibited biofilm formation. Unexpectedly, VTK‐LL37 showed inhibitory activity against *P. aeruginosa*.

In addition to enhancing the specific killing ability of peptides by adding target recognition sequences, amino acid substitutions may also have beneficial effects. For example, the specific killing ability of peptides against *P. aeruginosa* can be enhanced by tryptophan substitution^36^. In order to more systematically design specific targeting antimicrobial peptides, researchers develop the database filtering technique^37^. Based on analysis of the most likely parameters from the peptide library, antimicrobial peptides with efficient activity against methicillin‐resistant *S. aureus* were designed from scratch.

Previous studies have shown that most antimicrobial peptides accumulate on the surface of the bacterial membrane after being attracted to negatively charged components on the surface of pathogenic bacteria, mainly through electrostatic interactions. When antimicrobial peptides reach a certain threshold concentration on the surface of the cell membrane, they increase the permeability of the bacterial membrane through different modes of action, inducing lysis of the cell membrane and efflux of cell contents, thus causing bacterial death^38^.

LTA is a common negatively charged component on the surface of *S. aureus*. Therefore, we first explored its binding ability to antimicrobial peptides. The results showed that the ability of SFK2 to bind LTA was dose‐dependent. In addition, SFK2 had a greater ability to bind LTA than broad‐spectrum antimicrobial peptides at MICs^39^. We speculated that this may be an important factor in the ability of the peptide to specifically target *S. aureus*. Lipids are an important component of bacterial cell membranes and a common binding site for antimicrobial drugs to exert their antimicrobial effects. We then used POPG/CL at a mass ratio of 3:1 to simulate *S. aureus* bacterial membranes and further analyzed the interaction of SFK2 with lipids using microscale thermophoresis (MST) experiments. The results showed a typical S‐shaped binding curve. Liposomal calcium xanthophyll leakage experiments demonstrated that antimicrobial peptides had a greater affinity to bacterial membranes than to eukaryotic cell membranes, which is consistent with our conjecture^40^. To further test this hypothesis, we performed qualitative and quantitative analyses using laser confocal scanning microscopy and flow cytometry. Finally, the membrane‐damaging effects of SFK2 on *S. aureus* were directly observed using SEM and TEM.

It is worth considering whether antimicrobial peptides lose their activity due to various factors in the in vivo environment. Therefore, in vivo animal testing is often used to assess the practical applications of antimicrobial peptides. Researchers found that the peptide showed good therapeutic efficacy in treating a skin infection model established by *S. aureus* ^41^ owing to the strong antibacterial activity and targeting ability against *S. aureus*. We constructed and evaluated a mouse model of systemic infection using intraperitoneal and tail vein injections. Both modes of administration significantly reduced the number of colonies in the organs, and symptoms such as hepatocellular rupture and inflammatory cell infiltration were drastically reduced compared with those in the saline control. Because piglets are similar to humans in terms of anatomy, physiology, and nutritional metabolism, we further validated the in vivo effects of SFK2 using piglets as a model (Figure 7). It was found that SFK2 remained active in vivo in a piglet model for treating *S. aureus* infection. These results indicate that the heptapeptide sequence screened using phage display technology can effectively improve the specific targeting ability of the peptide against *S. aureus*, providing a reference for the subsequent development of narrow‐spectrum antibacterial drugs.

**Figure 8:**
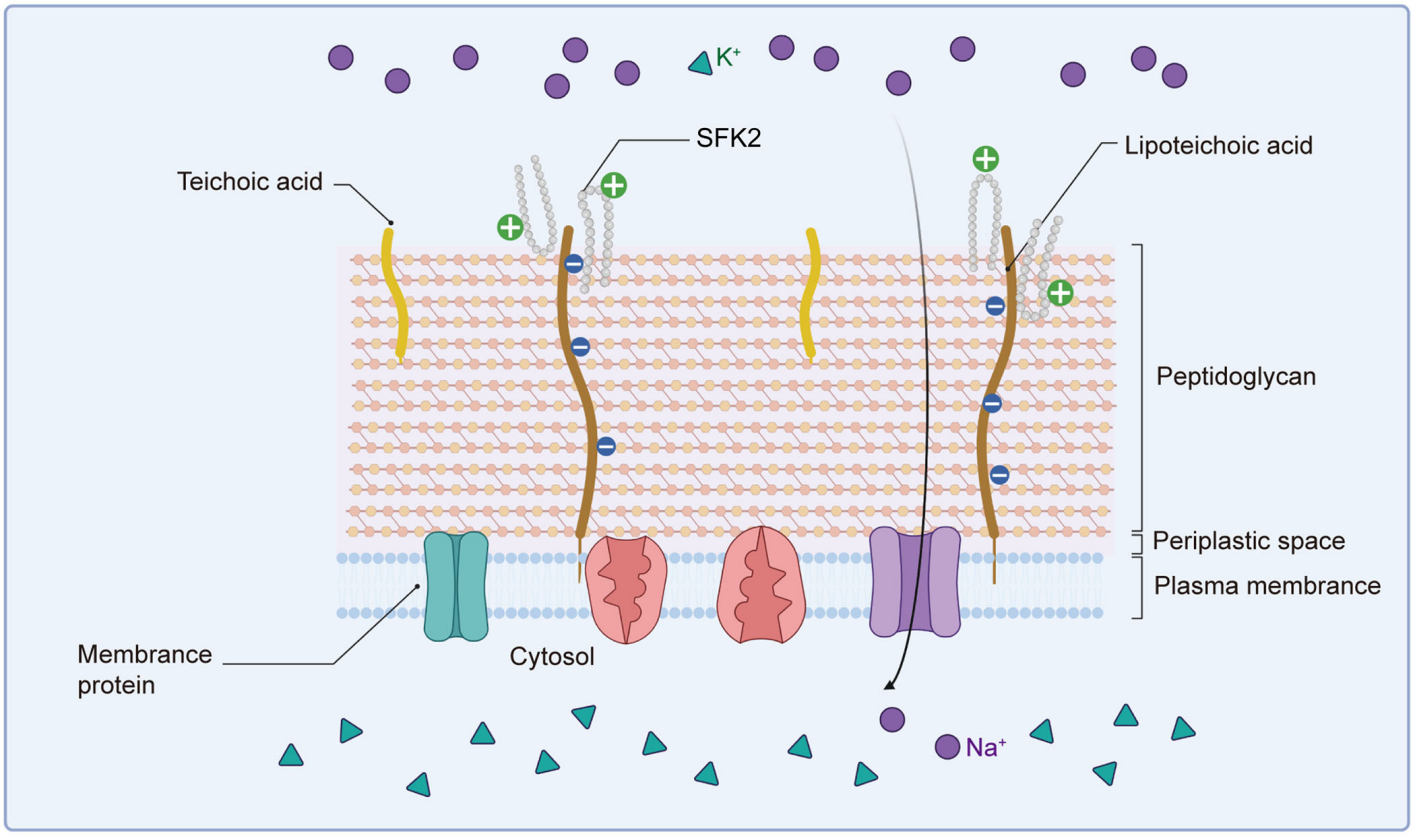
Schematic diagram of how SFK2 kills *Staphylococcus aureus*. Positively charged SFK2 binds to negatively charged LTA through electrostatic interaction, inducing depolarization of the cytoplasmic membrane, and thus exerting antibacterial effects.

In this study, we screened the heptapeptide sequence using phage display technology and concatenated it with Temporin‐SHf to obtain a chimeric peptide. The results of the antibacterial mechanism showed that SFK2 first bound to LTA on the surface of *S. aureus* through electrostatic effects, causing depolarization of the cytoplasmic membrane, resulting in severe damage to the cell membrane and the outflow of cell contents to exert antibacterial effects (Figure 8). Additionally, SFK2 showed good biocompatibility and in vivo therapeutic effects in mice and piglet. Our study provides potential antimicrobial peptides for targeted therapy of pathogenic bacteria.

## MATERIALS AND METHODS

### Microorganisms and cell lines

The strains *S. aureus* ATCC 6538, *P. aeruginosa* 27853, *E. coli* ATCC 25922, *P. aeruginosa* ATCC 15442, and *S. epidermidis* ATCC12228 were purchased from Shanghai Luwei Technology Co. Ltd. The strains *S. aureus* CVCC1882, *E. coli* K88, and *E. coli* K99 were kindly provided by the China Veterinary Culture Collection Center. Human embryonic kidney (HEK) 293 T cells were obtained from the College of Animal Science and Technology, China Agricultural University. Blood cells were obtained from healthy female Doric × Landrace × Large White pigs.

### Surface panning procedure of the phage display library

The Ph.D.^TM^‐7 phage display peptide library (New England Biolabs) was biopanned to identify *S. aureus*‐binding phages. Whole cells of *S. aureus* ATCC 6538 were prepared using PBS (10 mM, pH = 7.4) and resuspended overnight in a 96‐well plate at 4°C. Then, the solution was poured out, and the container was filled with blocking buffer and incubated at 4°C for 1 h. Next, the blocking buffer was discarded, and the 96‐well plates were washed six times quickly with TBST (TBS + 0.1% [v/v] Tween‐20). The phage library with a final concentration of 1.0 × 10^13^ pfu/ml was transferred to the coated plate and gently shaken for 60 min at room temperature. Subsequently, unbound phages were removed, and the plate was washed 10 times with TBST. After adding 100 μl of elution buffer (0.2 M Glycine‐HCl, pH = 2.2) to the plates, bound phages were obtained and transferred to sterilized microcentrifuge tubes. The eluate was added to 20 ml of *E. coli* 2738 culture medium and incubated for 4.5 h at 37°C with vigorously shaking for amplification. Afterward, 1/6 volume of 20% PEG/2.5 M NaCl was added to the eluate, and the pellet obtained by centrifugation (4°C, 12,000*g*) for 10 min was resuspended in 200 μl of TBS. The amplified eluate was titrated on LB/IPTG/X‐Gal plates to determine the titer of the phage. The concentration of Tween‐20 in TBST was adjusted to 0.5% (v/v), and the second and third rounds of surface washing were performed as described above.

### Extraction and sequencing of phage DNA

The phage eluent obtained in the third round of biological elution was amplified and titrated on an LB/IPTG/X‐Gal plate. Twenty‐four blue plaques were randomly picked from the titration plate with the tip of a straw and transferred to a test tube containing a diluted *E. coli* 2738 culture for amplification. After centrifugation at 14,000 rpm for 30 s, 500 μl of the supernatant was transferred to a new centrifuge tube, and 200 μl of 20% PEG/2.5 M NaCl was added to stand for 20 min. The supernatant was centrifugated at 14,000 rpm at 4°C for 10 min and then discarded. The phage pellets were suspended in 100 μl of KI buffer and incubated at room temperature for 20 min after adding 250 μl of ethanol. The supernatant was discarded after centrifugation, and the pellet was washed with 70% ethanol (0.5 ml) and centrifuged again to dry the particles briefly under vacuum. Finally, the pellets were suspended in 30 μl of TE buffer and sequenced.

### Determination of affinity between peptides and *S. aureus* using ELISA

Intact *S. aureus* ATCC 6538 cells were prepared with PBS (10 mM, pH = 7.4) and suspended overnight in an ELISA plate at 4°C. After shaking out the excess target solution, the blocking buffer was loaded into the culture plate and cultured at 4°C for 2 h. The blocking buffer was removed, and each plate was washed six times with TBST (TBS + 0.5% [v/v] Tween‐20). The diluted phage was transferred to a plate coated with target molecules, shaken at room temperature for 2 h, and washed six times with TBST. Finally, the streptavidin–HRP method was used to detect binding, and the TMB substrate was used to develop the color. Absorbance was recorded at 450 nm using a microplate reader.

### Peptide synthesis

The designed peptides were synthesized by GL Biochem. The polypeptide was synthesized using the Fmoc solid‐phase method and purified using reversed‐phase high‐performance liquid chromatography. The purity of the samples was greater than 95%. The relative molecular weight of the peptide was determined using matrix‐assisted laser desorption/ionization time‐of‐flight mass spectrometry, and the results were close to the theoretical relative molecular weight.

### Assay of the antimicrobial activity of peptides

MICs were determined to evaluate the antimicrobial activities of the peptides^42^. The bacteria were inoculated into Mueller‐Hinton broth (MHB) medium in advance and placed in a shaker at 37°C until the logarithmic growth phase. The optical density value at OD_600_ of the bacterial suspension was adjusted to 0.4, and then diluted 1000 times with sterile MHB medium. Subsequently, the peptides with concentrations of 0.25–128 μM were prepared using bovine serum albumin (BSA) solution (0.2% BSA and 0.01% acetic acid) as the solvent on a 96‐well plate. Different concentrations of peptides and bacterial liquids were mixed in equal volumes. MHB medium containing bacteria was used as a positive control. MHB medium without bacteria was used as a negative control. Then, the mixture of peptides and bacteria was cultured in an incubator at 37°C for 18–24 h. The optical density of each well was measured at 492 nm using a microplate reader. The lowest drug concentration corresponding to a culture mixture with an optical density of less than 0.1 is the MIC of the peptide. The assay was repeated three times in duplicate.

### Hemolytic activity assay

The hemolytic activity of the peptide was calculated by measuring the level of protein released from mammalian red blood cell lysis induced by the peptide^43^, ^44^. Fresh porcine blood was collected from the jugular vein and washed with PBS three times, and red blood cells were collected. A red blood cell suspension of 10% (v/v) concentration was prepared in 10 mM PBS (pH = 7.4). After mixing with different concentrations of peptides in equal volume, they were incubated at 37°C for 2 h. Red blood cells treated with PBS and 0.1% Triton X‐100 were used as negative and positive controls, respectively. The assay was repeated three times. After the sample was centrifuged, the supernatant was transferred to a new 96‐well plate, and the optical density at 570 nm was measured using a microplate reader. Hemolysis was calculated as follows:

$$\text{Hemolytic activity}\left(\%\right)=\frac{{\text{OD}}_{\text{test}\;\mathrm{sample}}-{\mathrm{OD}}_{\text{negative control}}}{{\text{OD}}_{\text{positive control}}-{\mathrm{OD}}_{\text{negative control}}}\times 100\%$$

### Cytotoxicity measurement

HEK 293 T cells were used to detect peptide cytotoxicity using MTT dye^45^. The cells were diluted with DMEM to adjust the final concentration to 2 × 10^5^ cells/ml, which was added to 96‐well plates and cultured in a CO_2_ incubator at 37°C for 24 h. After removing the supernatant, the peptides with final concentrations of 0.25–128 μM were added to the 96‐well plate and incubated in the incubator for 2 h. Cells without polypeptides were used as positive controls, and DMEM was used as a negative control. Subsequently, 20 μl of MTT (5 mg/ml) was added to all the reaction systems and incubated for another 2 h at 37°C. After that, the supernatant was discarded, 150 μl of dimethyl sulfoxide (DMSO) was added to each well, and the 96‐well plate was placed on a shaker oscillator for 10 min to fully dissolve the formazan crystals. The absorbance of each well was measured at 570 nm using a microplate reader. The test was repeated three times.

### Salt sensitivity assays

The assay was performed to analyze the salt ion stability of SFK2 by measuring changes in MIC values in different physiological salt concentrations^38^, ^46^. Different concentrations of salts (0.3 M NaCl, 9 × 10^−3 ^M KCl, 2 × 10^−3 ^M MgCl_2_, 1.6 × 10^−5 ^M ZnCl_2_, 1.2 × 10^−5 ^M NH_4_Cl, and 6 × 10^−6 ^M FeCl_3_) were dissolved in BSA diluent, and the next steps were the same as those for the determination of MIC.

### Circular dichroism spectroscopy analysis

Assays were performed using circular dichroism spectrometry to analyze the secondary structure of the peptides^47^. The peptides were dissolved in 10 mM PBS (10 mM, pH = 7.4), 30 mM SDS, and 50% TFE solution so that the final concentration of the peptides was 50 mM. The ellipticity of the peptide in the wavelength range of 190–250 nm was recorded using a spectropolarimeter. Subsequently, the average residual ellipticity (*θ*_M_) was calculated according to the following formula:

$$\theta_{M}=\frac{1000\times \theta_{\mathrm{obs}}}{c\times l\times n}$$

where *θ*_obs_ is the observed ellipticity; *c*, *l*, and *n* are the concentration of peptide, the path length, and the number of amino acids, respectively.

### Bactericidal kinetics

The antibacterial efficiency of SFK2 was evaluated by measuring its bactericidal kinetics against *S. aureus* ^48^. The suspension concentration of *S. aureus* ATCC 6538 in the logarithmic growth phase was adjusted to 10^6^ CFU/ml and then treated with peptide solutions at 1 ×, 2 ×, and 4 × MICs. The mixtures were diluted with PBS and inoculated onto Mueller Hinton Agar plates at different intervals (0, 3, 5, 15, and 30 min and 1 and 2 h). The Petri dishes were incubated at 37°C for 24 h, and the bacterial colonies were counted.

### LTA binding assay

The ability of the peptides to bind to LTA was detected using the BODIPY‐TR‐cadaverine (BC) fluorescent probe substitution method^49^. BC fluorescent probe and LTA from *S. aureus* were mixed in Tris‐HCl buffer and incubated in an incubator at 37°C for 4 h. Peptide solutions of different concentrations (0.25–128 µM) were prepared in 96‐well plates, and equal volumes of BC‐LTA buffer were mixed with the peptide solutions and incubated at 37°C for 1 h. Polymyxin B (100 μg/ml) was used as a positive control, and no peptide addition was used as a negative control. Each sample was analyzed three times. Subsequently, the fluorescence of each sample was measured using a microplate reader at an excitation wavelength of 580 nm and an emission wavelength of 620 nm.

### Analysis of peptide–liposome interactions using MST

Membranes of *S. aureus* were simulated using POPG/CL^50^. POPG/CL at a mass ratio of 3:1 was dissolved in chloroform and dried with nitrogen, and then PBST was added to obtain a final concentration of 3 mM, followed by ultrasound treatment for 10 min. The FITC‐labeled peptides were dissolved in PBST, and 16 liposomes were prepared at different dilutions (maximum concentration of 1.5 mM). After mixing the FITC‐labeled peptides with liposomes at different dilutions in equal volumes, the mixture was aspirated using a standard glass capillary tube and assayed using the Monolith NT.115 system^40^.

### Cytoplasmic membrane depolarization assay

The fluorescent probe DiSC3(5) was used to assess the changes in the cytoplasmic membrane permeability of *S. aureus* treated with peptides^8^. *S. aureus* ATCC 6538 in the logarithmic phase was centrifuged and washed with HEPES buffer (pH = 7.4, containing 40 mM glucose) three times to prepare the bacterial suspension (OD_600_ = 0.1). Subsequently, DiSC3‐5 and KCl solutions with final concentrations of 0.4 μM and 200 mM, respectively, were mixed with the bacterial suspension and incubated at 37°C for 1.5 h. The basic fluorescence values of the bacterial solutions were determined at excitation and emission wavelengths of 622 and 670 nm, respectively. Changes in fluorescence intensity were recorded after adding different concentrations of peptide solutions (1 × MIC and 2 × MIC).

### Membrane integrity observation

Confocal laser scanning microscopy was used to assess the membrane integrity of *S. aureus* ATCC 6538 after incubation with peptides^51^ *. S. aureus* ATCC 6538 in the logarithmic growth phase was collected by centrifugation, and a bacterial suspension (OD_600_ = 0.05) was prepared in PBS. The final concentration of 8 μM peptide was mixed with the same volume of cell suspension, and then 2.5 μM SYTO9 and 50 μg/ml PI dye were added. The mixed solution was then incubated at 37°C for 2 h. The samples were imaged using a confocal laser scanning microscope. A peptide‐free mixture was used as a control.

To investigate the targeting ability of SFK2, *S. aureus* ATCC 6538 and *E. coli* ATCC 25922 were mixed 1:1 and treated with 8 μM of SFK2. SYTO9 and PI fluorescent dyes were added, and the cells were observed using laser scanning confocal microscopy.

### Co‐localization of FITC‐labeled peptides with dead bacteria

FITC‐labeled SFK2 was used to determine the site of action against *S. aureus* 6538. *S. aureus* 6538 in the logarithmic growth phase was collected by centrifugation, and the bacterial suspension was prepared in PBS (OD_600_ = 0.1). A final concentration of 8 µM FITC‐SFK2 was mixed with the same volume of bacterial suspension, and 50 µg/mg of PI dye was added. The mixture was incubated at 37°C away from light for 2 h. The samples were imaged using a laser confocal scanning microscope.

### Flow cytometry

This assay was performed using flow cytometry to quantify the membrane integrity of bacteria after peptide treatment^52^. *S. aureus* ATCC 6538 in the logarithmic growth phase was collected by centrifugation and diluted with PBS (10 mM, pH = 7.4) to prepare a bacterial suspension (OD_600_ = 0.1). Peptide solutions in the concentration range of 4–16 µM were prepared on 96‐well plates. The bacterial dilutions were mixed with the peptide solution in equal volumes and incubated at 37°C for 1 h. Next, the PI fluorescent dye with a final concentration of 10 μg/ml was added to the mixture to continue to incubate for 30 min. The fluorescence intensity of PI in different systems was measured using flow cytometry.

### SEM and TEM characterization

Suspensions of *S. aureus* ATCC 6538 (OD_600_ = 0.2) were prepared as described above. Afterward, peptides with a final concentration of 1 × MIC were added to the bacterial suspension and incubated for 2 h at 37°C. The bacterial suspension was centrifuged, the supernatant was discarded, and 1 ml of electron microscope fixative was added and fixed at 4°C for 24 h^53^.

The samples were dehydrated in gradients of 30%, 50%, 70%, 85%, 95%, and 100% alcohol and transferred to 50% and 100% tert‐butanol solutions for further processing. Bacteria were collected after centrifugation for CO_2_ critical point drying. The bacteria were subsequently fixed on a SEM sample stage and observed using SEM after gold centrifugal sputtering.

For TEM, the samples were dehydrated in gradients of 30%, 50%, 70%, 85%, 95%, and 100% alcohol, followed by 50% and 100% propanol. After centrifugation, the bacteria were collected and embedded in epoxy resin. Ultrathin sections were prepared, stained with uranyl acetate and lead citrate, and observed using TEM.

### Drug resistance assay

The fold change in the MIC of peptides and ciprofloxacin against *S. aureus* ATCC 6538 was measured using the continuous passaging method to evaluate the development of drug resistance^3^. The MIC values of the peptide and vancomycin against *S. aureus* ATCC 6538 were determined according to the above method, and the solutions in the sub‐MIC wells were aspirated and transferred to a fresh MHB medium for the next round of MIC determination. The procedure lasted for 30 days.

### Biosafety evaluation

Eighteen male BALB/c mice (weighing approximately 30 g) were randomly divided into three groups (control, low‐dose, and high‐dose groups). Mice in the low‐ and high‐dose groups were intraperitoneally injected with 15 and 30 mg/kg polypeptide solutions, respectively, once daily. Mice in the control group were injected intraperitoneally with 150 µl of saline once daily. The experiment lasted for 7 days, and the changes in the body weight of the mice were recorded. After the seventh intraperitoneal injection for another 12 h, the mice were anesthetized with ether and euthanized. Blood was collected from the orbital vein, and serum was analyzed by biochemical analysis after centrifugation. The livers and kidneys of mice were collected and weighed. Afterward, the organs were fixed in a 4% paraformaldehyde solution and stained with H&E for analysis.

Twelve male BALB/c mice (weighing approximately 20 g) were randomly divided into two groups (control and peptide‐treated groups). Mice in the peptide‐treated group were injected intravenously into the tail vein with 5 mg/kg peptide solution once daily. In the control group, mice were injected once daily with saline via the tail vein. The experiment was continued for 7 days, and changes in body weight were recorded. Mice were anesthetized with ether and euthanized 12 h after the seventh tail vein injection. Blood was collected from the orbital vein and centrifuged, and the serum was analyzed biochemically. The livers and kidneys of mice were collected and weighed. The organs were then fixed in a 4% paraformaldehyde solution and analyzed by H&E staining.

### Analysis of antibacterial activity in vivo

Eighteen male BALB/c mice (weighing approximately 30 g) were randomly divided into three groups (healthy, control, and treatment groups). The control and treatment groups were injected intraperitoneally with 400 μl of *S. aureus* ATCC 6538 bacterial suspension (OD_600_ = 1.3), and the healthy group was injected intraperitoneally with 400 μl of saline. Two hours after infection, the control and treatment groups were injected intraperitoneally with 150 μl of saline and peptide (15 mg/kg), respectively. The mice were anesthetized with ether after 12 h and killed. The livers, kidneys, spleens, and lungs of the mice were collected and weighed for quantitative bacterial analysis. The remaining fraction was fixed in 4% paraformaldehyde and analyzed by H&E staining.

Eighteen male BALB/c mice (weighing approximately 20 g) were randomly divided into three groups (healthy, control, and treatment groups). The control and treatment groups were injected intraperitoneally with 400 μl of *S. aureus* ATCC 6538 bacterial suspension (OD_600_ = 1.3), and the healthy group was injected intraperitoneally with 400 μl of saline. Two hours after infection, the control and treatment groups were injected with saline and peptide (5 mg/kg), respectively, into the tail vein. Twelve hours later, the mice were anesthetized with ether and euthanized. The livers, kidneys, spleens, and lungs of the mice were collected, weighed, and analyzed for bacterial quantification. The remaining parts were fixed with a 4% paraformaldehyde solution and stained with H&E for analysis.

Fifteen male Dorec × Landrace × Large White weaned piglets were randomized into three groups (healthy, control, and treatment groups). In the control and treatment groups, piglets were intraperitoneally injected with 50 ml of *S. aureus* 6538 suspension (OD_600_ = 1.5). Piglets in the healthy group were intraperitoneally injected with 50 ml of saline. Two hours later, piglets in the control and treatment groups were injected with 10 ml of saline and polypeptide (3 mg/kg), respectively. All piglets were killed 12 h after treatment. The livers, kidneys, spleens, and lungs of the piglets were collected and weighed for colony counting. The remaining tissues were fixed in a 4% paraformaldehyde solution for histological observation using H&E staining.

### Statistical analysis

Data are described as mean ± standard deviation. Data were analyzed using SPSS 22 for one‐way analysis of variance and Duncan's method for multiple comparisons. Results were plotted using GraphPad Prism 8.

## AUTHOR CONTRIBUTIONS

**Tao Wang**: Data curation (lead); writing—original draft (lead). **Peng Tan**: Writing—review and editing (equal). **Qi Tang**: Writing—review and editing (equal). **Chenlong Zhou**: Writing—review and editing (equal). **Yakun Ding**: Conceptualization (equal). **Shenrui Xu**: Conceptualization (equal). **Mengda Song**: Conceptualization (equal). **Huiyang Fu**: Conceptualization (equal). **Yucheng Zhang**: Conceptualization (equal). **Xiaohui Zhang**: Conceptualization (equal). **Yueyu Bai**: Conceptualization (equal). **Zhihong Sun**: Conceptualization (equal); funding acquisition (equal); project administration (equal); resources (supporting). **Xi Ma**: Funding acquisition (lead); project administration (lead); writing—original draft (lead); writing—review and editing (lead).

## ETHICS STATEMENT

All 12‐week‐old BALB/c male mice (weighing approximately 30 g) and 8‐week‐old BALB/c male mice (weighing approximately 20 g) were provided by Beijing Spelford Biotechnology Co. All of the 3‐week‐old Dorec × Landrace × Large White weaned piglets (weighing about 6 kg) were supplied by Chongqing Hechuan Kangde Pig Raising Co. Experimental animals were housed and handled in accordance with Chinese laws and guidelines (Protocol GK‐FCZ2001545), EU Directive 2010/63/EU Animal Experimentation, and the Regulations for the Protection of Animals for Scientific Use at China Agricultural University (2010‐SYXK‐0037). All protocols were approved by the Animal Protection and Utilization Committee of China Agricultural University (AW90303202‐1‐1, AW50204202‐2‐8, and AW50204202‐2‐9).

## CONFLICT OF INTERESTS

The authors declare no conflict of interests.

## Supporting information

Supporting information.

## Data Availability

The data are included in this article and supporting information.
