# Supplementary material for: Phage‐displayed heptapeptide sequence conjugation significantly improves the specific targeting ability of antimicrobial peptides against Staphylococcus aureus
Source: mLife. 2024 May 27;3(2):251–68. doi: 10.1002/mlf2.12123 (PMC11211671; doi:10.1002/mlf2.12123)
Supplement: Supplementary file 1 — Supporting information. [file MLF2-3-251-s001.docx]

**Phage-displayed heptapeptide sequence conjugation significantly improves the specific targeting ability of antimicrobial peptides against *Staphylococcus aureus***

*Tao Wang ^a, b^, Peng Tan ^a^, Qi Tang ^a^, Chenlong Zhou ^a^, Yakun Ding ^c^, Shenrui Xu ^a^, Mengda Song ^c^, Huiyang Fu ^a^, Yucheng Zhang ^a^, Xiaohui Zhang ^b^, Yueyu Bai ^c, d^, Zhihong Sun ^e, *^,* and *Xi Ma ^a, *^*

*^a^ State Key Laboratory of Animal Nutrition and Feeding, College of Animal Science and Technology, China Agricultural University, Beijing, 100193, China.*

*^b^* *Luoyang Key Laboratory of Animal Genetic and Breeding, College of Animal Science, Henan University of Science and Technology, Luoyang, Henan, 471000, China.*

*^c^* *Key Laboratory of Innovative Utilization of Indigenous Cattle and Sheep Germplasm Resources (Co-construction by Ministry and Province), Ministry of Agriculture and Rural Affairs, Zhengzhou University, Zhengzhou, 450001, China.*

*^d^ Animal Health Supervision in Henan Province, Zhengzhou, Henan, 450003, China.*

*^e^ Laboratory for Bio-Feed and Molecular Nutrition, Department of Animal*

*Science and Technology, Southwest University, Chongqing, China*

**^*^ *Corresponding author.***

*E-mail address: sunzh2002cn@aliyun.com (ZH. Sun), maxi@cau.edu.cn (X. Ma)*

**Table S1** Phage monoclonal after three rounds of screening

| Round of screening | Input phage (pfu/mL) | Output phage (pfu/mL) | Yield |
| --- | --- | --- | --- |
| 1 | 1.0×10^13^ | 2.09×10^6^ | 2.09×10^-7^ |
| 2 | 2.57×10^12^ | 3×10^4^ | 1.17×10^-8^ |
| 3 | 4×10^14^ | 2.54×10^7^ | 6.35×10^-8^ |

**Table S2** DNA sequences of phage clones

| No. | DNA sequence |
| --- | --- |
| 1 | CGGCCGAACCTCCACCATGCTTATGCAGCTTCAGATGAGAGTGAGAATAGAAAGGTACC |
| 2 | CGGCCGAACCTCCACCCTGCGTATGCCTATGATGCATAGAGTGAGAATAGAAAGGTACC |
| 3 | CGGCCGAACCTCCACCATGCTTATGAGGCTTATGAAAAGAGTGAGAATAGAAAGGTACC |
| 4 | CGGCCGAACCTCCACCATGCTTATGATGAAAAGGCCAAGAGTGAGAATAGAAAGGTACC |
| 5 | CGGCCGAACCTCCACCAGGCAGAGTCCGATGCGAAATAGAGTGAGAATAGAAAGGTACC |
| 6 | CGGCCGAACCTCCCCCATGCTTATGAGGCTTATGAAAAGAGTGAGAATAGAAAGGTACC |
| 7 | CGGCCGAACCTCCACCCTGCGTATGCCTATGATGCATAGAGTGAGAATAGAAAGGTACC |
| 8 | CGGCCGAACCTCCACCACGCTTAGGAATCTTATGCCAAGAGTGAGAATAGAAAGGTACC |
| 9 | CGGCCGAACCTCCACCATGATGATGATGCCGCACAAAAGAGTGAGAATAGAAAGGTACC |
| 10 | CGGCCGAACCTCCACCATGATGATGATGCCGCACAAAAGAGTGAGAATAGAAAGGTACC |
| 11 | CGGCCGAACCTCCACCAAGCCTATAATGCTTATGATGAGAGTGAGAATAGAAAGGTACC |
| 12 | CGGCCGAACCTCCACCCTGCGTATGCCTATGATGCATAGAGTGAGAATAGAAAGGTACC |
| 13 | CGGCCGAACCTCCACCATGCTTATGAGGCTTATGAAAAGAGTGAGAATAGAAAGGTACC |
| 14 | CGGCCGAACCTCCACCATGCTTATGCAGCTTCAGATGAGAGTGAGAATAGAAAGGTACC |
| 15 | CGGCCGAACCTCCCCCCCGCTTATCAGGCTTATGACCAGAGTGAGAATAGAAAGGTACC |
| 16 | CGGCCGAACCTCCACCATACGTATGCGGCCTATTAACAGAGTGAGAATAGAAAGGTACC |
| 17 | CGGCCGAACCTCCACCCGACGCCCGAACCCAATACGAAGAGTGAGAATAGAAAGGTACC |
| 18 | CGGCCGAACCTCCACCCAGAGCCGTAGGCTCAGCCTTAGAGTGAGAATAGAAAGGTACC |
| 19 | CGGCCGAACCTCCACCATGATGACGATGCCAACTCCAAGAGTGAGAATAGAAAGGTACC |
| 20 | CGGCCGAACCTCCACCACGCATCCAAGACTTATGATGAGAGTGAGAATAGAAAGGTACC |
| 21 | CGGCCGAACCTCCACCCCCAAGCATCCTAGTCACATAAGAGTGAGAATAGAAAGGTACC |
| 22 | CGGCCGAACCTCCACCATGCTTATGAGGCTTATGAAAAGAGTGAGAATAGAAAGGTACC |
| 23 | CGGCCGAACCTCCACCCTGCGTATGCCTATGATGCATAGAGTGAGAATAGAAAGGTACC |
| 24 | CGGCCGAACCTCCACCCGTCGCACCATCAAGCGCATGAGAGTGAGAATAGAAAGGTACC |

**Table S3** DNA sequence of the phage clones and heptapeptide sequence

| Number | Sequence | Heptapeptide sequence | Frequency |
| --- | --- | --- | --- |
| 2,7,12,13,23 | CTGCGTATGCCTATGATGCAT | MHHRHTQ | 5 |
| 3,6,22 | ATGCTTATGAGGCTTATGAAA | FHKPHKH | 3 |
| 1,14 | ATGCTTATGCAGCTTCAGATG | HLKLHKH | 2 |
| 9,10 | ATGATGATGATGCCGCACAAA | FLRHHHH | 2 |
| 20,21 | ACGCATCCAAGACTTATGATG | HHKSWMR | 2 |
| 5 | AGGCAGAGTCCGATGCGAAAT | NSHRTLP | 1 |
| 8 | ACGCTTAGGAATCTTATGCCA | WHKIPNR | 1 |
| 11 | AAGCCTATAATGCTTATGATG | HHKHYKL | 1 |
| 17 | CGACGCCCGAACCCAATACG | SYWVRAS | 1 |

**Table S4** Sequence and physicochemical parameters of antimicrobial peptides

| Peptides | Sequence | MW | Net charge | Hydrophobicity^a^ |
| --- | --- | --- | --- | --- |
| SF | SYWVRASGGGFFFLSRIF-NH_2_ | 2096.39 | +3 | 0.762 |
| SFR | SYWVRASGGGFFFLSRIFR-NH_2_ | 2252.58 | +4 | 0.673 |
| SFK | SYWVRASGGGFFFLSRIFK-NH_2_ | 2224.56 | +4 | 0.674 |
| SFR2 | SYWVRASGGGFFFLSRIFRR-NH_2_ | 2408.76 | +5 | 0.593 |
| SFK2 | SYWVRASGGGFFFLSRIFKK-NH_2_ | 2352.73 | +5 | 0.595 |
| SFR3 | SYWVRASGGGFFFLSRIFRRR-NH_2_ | 2564.95 | +6 | 0.520 |
| SFK3 | SYWVRASGGGFFFLSRIFKKK-NH_2_ | 2480.91 | +6 | 0.523 |

^a^Hydrophobicity is calculated by https://heliquest.ipmc.cnrs.fr/cgi-bin/ComputParams.py.

**Table S5** Hemolytic activity of peptides

| Peptides | MHC | Hemolysis (%) |
| --- | --- | --- |
| SF | ＞128 | 1.52±0.68 |
| SFR | ＞128 | 4.27±0.37 |
| SFK | ＞128 | 4.27±0.37 |
| SFR2 | ＞128 | 2.74±0.49 |
| SFK2 | ＞128 | 2.41±0.38 |
| SFR3 | ＞128 | 1.59±0.51 |
| SFK3 | ＞128 | 5.97±0.80 |

**Table S6** The MHC (μM), GM (μM), SI, and TI values of the peptides

| Peptides | MHC | GM | | SI | | TI |
| --- | --- | --- | --- | --- | --- | --- |
|  |  | *S. aureus* | Other bacteria | *S. aureus* | Other bacteria |  |
| SF | ＞128 | 5.657 | 128.000 | 45.255 | 2.000 | 0.044 |
| SFR | ＞128 | 2.000 | 80.635 | 128.000 | 3.175 | 0.025 |
| SFK | ＞128 | 2.828 | 90.510 | 90.510 | 2.828 | 0.031 |
| SFR2 | ＞128 | 2.000 | 90.510 | 128.000 | 2.828 | 0.022 |
| SFK2 | ＞128 | 2.000 | 128.000 | 128.000 | 2.000 | 0.016 |
| SFR3 | ＞128 | 8.000 | 101.594 | 32.000 | 2.520 | 0.079 |
| SFK3 | ＞128 | 1.414 | 64.000 | 181.047 | 4 | 0.022 |

**Table S7** The MIC values (µM) of SFK2 against *S. aureus* ATCC 6538 at physiological salt concentration

| Peptide | Control | NaCl^a^ | KCl^a^ | NH_4_Cl^a^ | MgCl_2_^a^ | CaCl_2_^a^ | ZnCl_2_^a^ | FeCl_3_^a^ |
| --- | --- | --- | --- | --- | --- | --- | --- | --- |
| SFK2 | 2 | 4 | 4 | 4 | 4 | 4 | 2 | 4 |


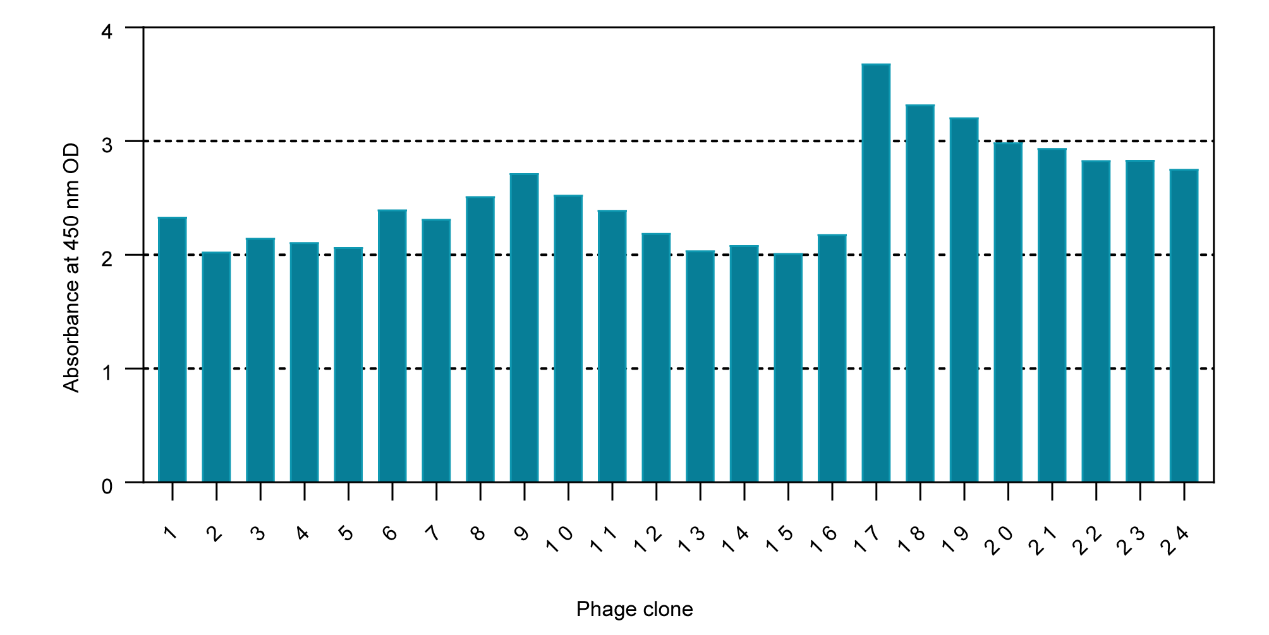


**Figure S1.** Phage monoclonal affinity assay results

SF: SYWVRASGGGFFFLSRIF-NH_2_

SFR: SYWVRASGGGFFFLSRIFR-NH_2_

SFK: SYWVRASGGGFFFLSRIFK-NH_2_

SFR2: SYWVRASGGGFFFLSRIFRR-NH_2_

SFK2: SYWVRASGGGFFFLSRIFKK-NH_2_

SFR3: SYWVRASGGGFFFLSRIFRRR-NH_2_

SFK3: SYWVRASGGGFFFLSRIFKKK-NH_2_

**Figure S2.** Chemical structural formula and sequence of the synthetic peptides.

Column: Gemini-NX 5μ C18 110A, 4.6*250mm

Solvent A: 0.1% Trifluoroacetic in 100% Acetonitrile

Solvent B: 0.1%Trifluoroacetic in 100% Water

Flow rate: 1.0 mL/min

Wavelength: 220 nm

Volume: 20 μL


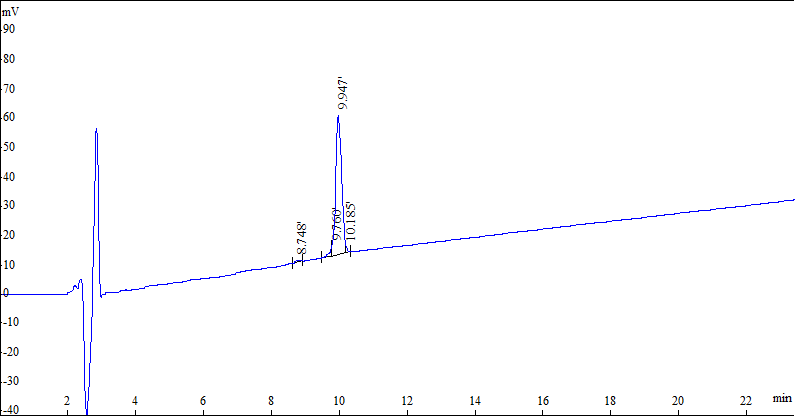


SF: SYVWRASGGGFFFLSRIF-NH_2_

| Rank | Time | Concentration | Area | Height |
| --- | --- | --- | --- | --- |
| 1 | 8.748 | 1.1217 | 6314 | 751 |
| 2 | 9.760 | 2.2236 | 12516 | 2991 |
| 3 | 9.947 | 95.8048 | 539258 | 47137 |
| 4 | 10.185 | 0.8499 | 4784 | 1848 |
| Total | - | 100 | 562872 | 52727 |

SFR: SYVWRASGGGFFFLSRIFR-NH_2_

| Rank | Time | Concentration | Area | Height |
| --- | --- | --- | --- | --- |
| 1 | 9.812 | 0.7313 | 12688 | 1981 |
| 2 | 10.667 | 0.4575 | 7938 | 1508 |
| 3 | 10.833 | 98.1871 | 1703462 | 218505 |
| 4 | 11.384 | 0.6241 | 10828 | 776 |
| Total | - | 100 | 1734916 | 222770 |


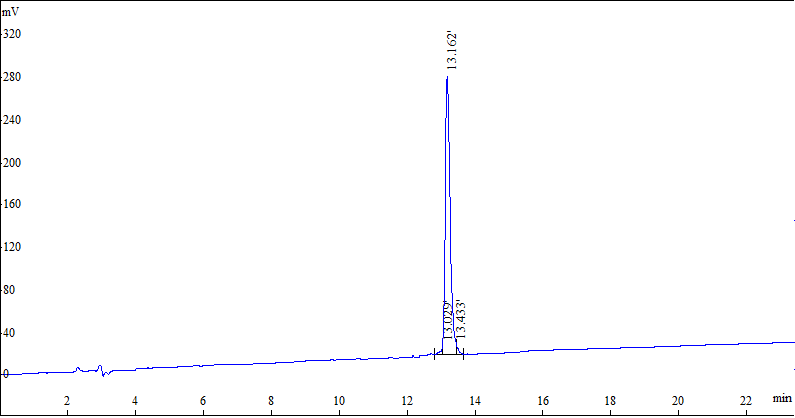


SFK: SYVWRASGGGFFFLSRIFK-NH_2_

| Rank | Time | Concentration | Area | Height |
| --- | --- | --- | --- | --- |
| 1 | 13.029 | 1.3488 | 34190 | 8078 |
| 2 | 13.162 | 97.3578 | 2467852 | 260959 |
| 3 | 13.433 | 1.2934 | 32785 | 8297 |
| Total | - | 100 | 2534827 | 277334 |


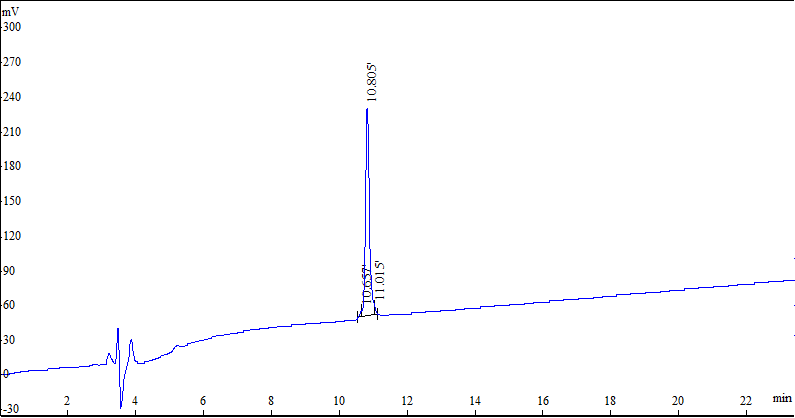


SFR2: SYVWRASGGGFFFLSRIFRR-NH_2_

| Rank | Time | Concentration | Area | Height |
| --- | --- | --- | --- | --- |
| 1 | 10.657 | 1.6888 | 22788 | 5419 |
| 2 | 10.805 | 97.0114 | 1309044 | 178695 |
| 3 | 11.015 | 1.2998 | 17539 | 7309 |
| Total | - | 100 | 1349371 | 191423 |

SFK2: SYVWRASGGGFFFLSRIFKK-NH_2_

| Rank | Time | Concentration | Area | Height |
| --- | --- | --- | --- | --- |
| 1 | 7.567 | 1.1019 | 147718 | 13177 |
| 2 | 10.615 | 1.3707 | 183743 | 9697 |
| 3 | 11.063 | 95.6235 | 12818752 | 851870 |
| 4 | 11.495 | 1.4635 | 196194 | 15954 |
| 5 | 11.941 | 0.4404 | 59034 | 3948 |
| Total | - | 100 | 13405441 | 894646 |


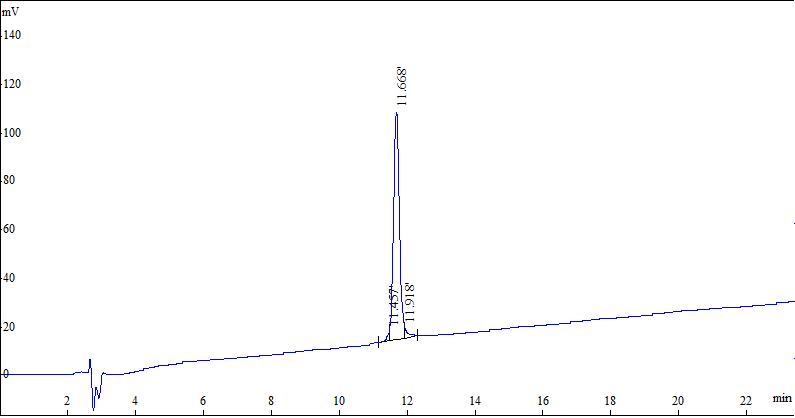


SFR3: SYVWRASGGGFFFLSRIFRRR-NH_2_

| Rank | Time | Concentration | Area | Height |
| --- | --- | --- | --- | --- |
| 1 | 11.457 | 1.3369 | 14779 | 4062 |
| 2 | 11.668 | 96.0143 | 1061427 | 93636 |
| 3 | 11.918 | 2.6488 | 29282 | 3984 |
| Total | - | 100 | 1105488 | 101682 |

SFK3: SYVWRASGGGFFFLSRIFKKK-NH_2_

| Rank | Time | Concentration | Area | Height |
| --- | --- | --- | --- | --- |
| 1 | 7.290 | 0.7631 | 36273 | 5013 |
| 2 | 10.816 | 0.7307 | 34735 | 6349 |
| 3 | 10.991 | 96.5621 | 4590209 | 550612 |
| 4 | 11.267 | 1.1895 | 56545 | 2802 |
| 5 | 11.288 | 0.2638 | 12538 | 2050 |
| 6 | 11.793 | 0.4908 | 23332 | 2375 |
| Total | - | 100 | 4753632 | 569201 |

**Figure S3.** Reversed-phase high-performance liquid-chromatography of synthetic peptides.

Instrument: Agilent-6125B

Probe: ESI

Nebulizer Gas Flow: 1.5 L/min

CDL: -20.0 v

CDL Temperature: 250℃

Block Temperature: 200℃

Probe Bias: +4.5kv

Detector: 1.5kv

T. Flow: 0.2mL/min

B. Conc.:50% H_2_O/50% ACN

SF: SYVWRASGGGFFFLSRIF-NH_2_

SFR: SYVWRASGGGFFFLSRIFR-NH_2_

SFK: SYVWRASGGGFFFLSRIFK-NH_2_

SFR2: SYVWRASGGGFFFLSRIFRR-NH_2_

SFK2: SYVWRASGGGFFFLSRIFKK-NH_2_

SFR3: SYVWRASGGGFFFLSRIFRRR-NH_2_

SFK3: SYVWRASGGGFFFLSRIFKKK-NH_2_

**Figure S4.** Matrix-assisted laser desorption/ionization time-of-flight mass spectrometry (MALDI-TOF MS) spectra of synthetic peptides.


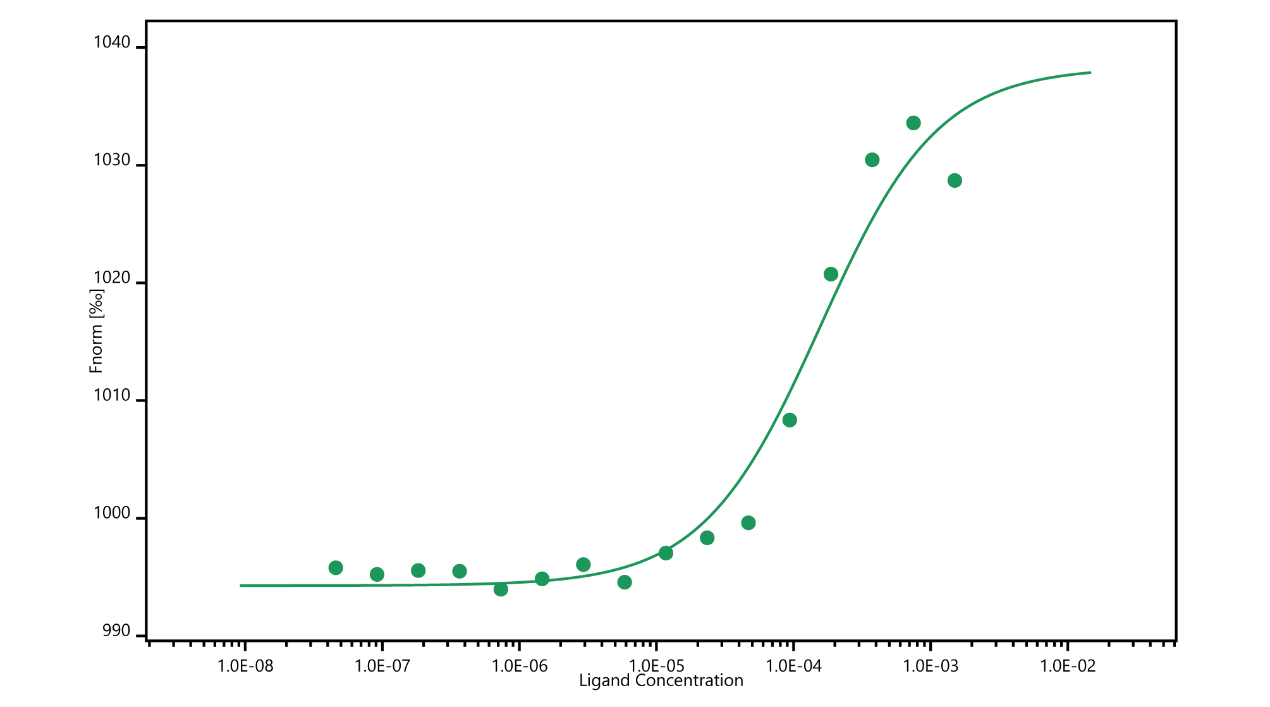


**Figure S5.** The degree of thermophoresis FITC-SFK2 upon binding to liposomes.


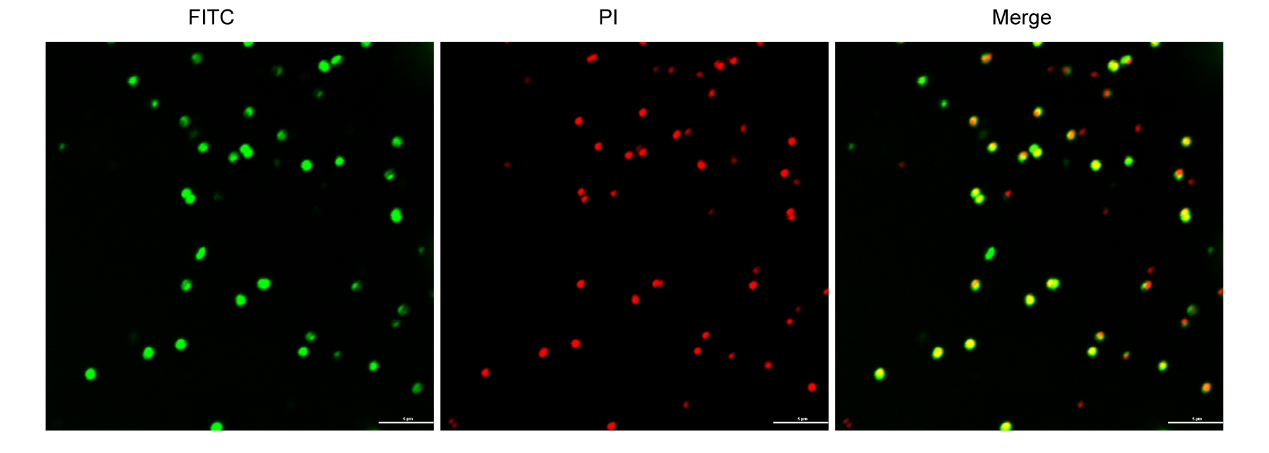


**Figure S6.** Laser confocal microscopy image analysis of FITC-labelled SFK2 with PI-treated *S. aureus* 6538. Scale bar : 5 μm.


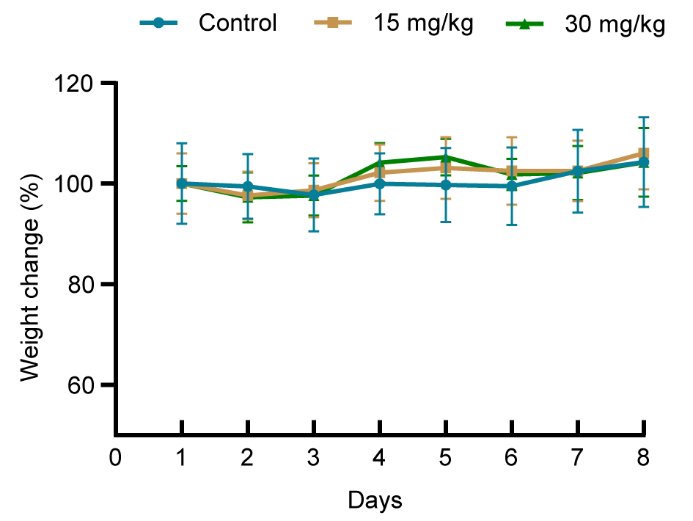


**Figure S7.** Body weight changes of BALB/C mice treated with different doses of SFK2. Weight change (%) = Weight of mice / Initial average weight × 100%. Data are expressed as mean ± standard deviation, n = 6.


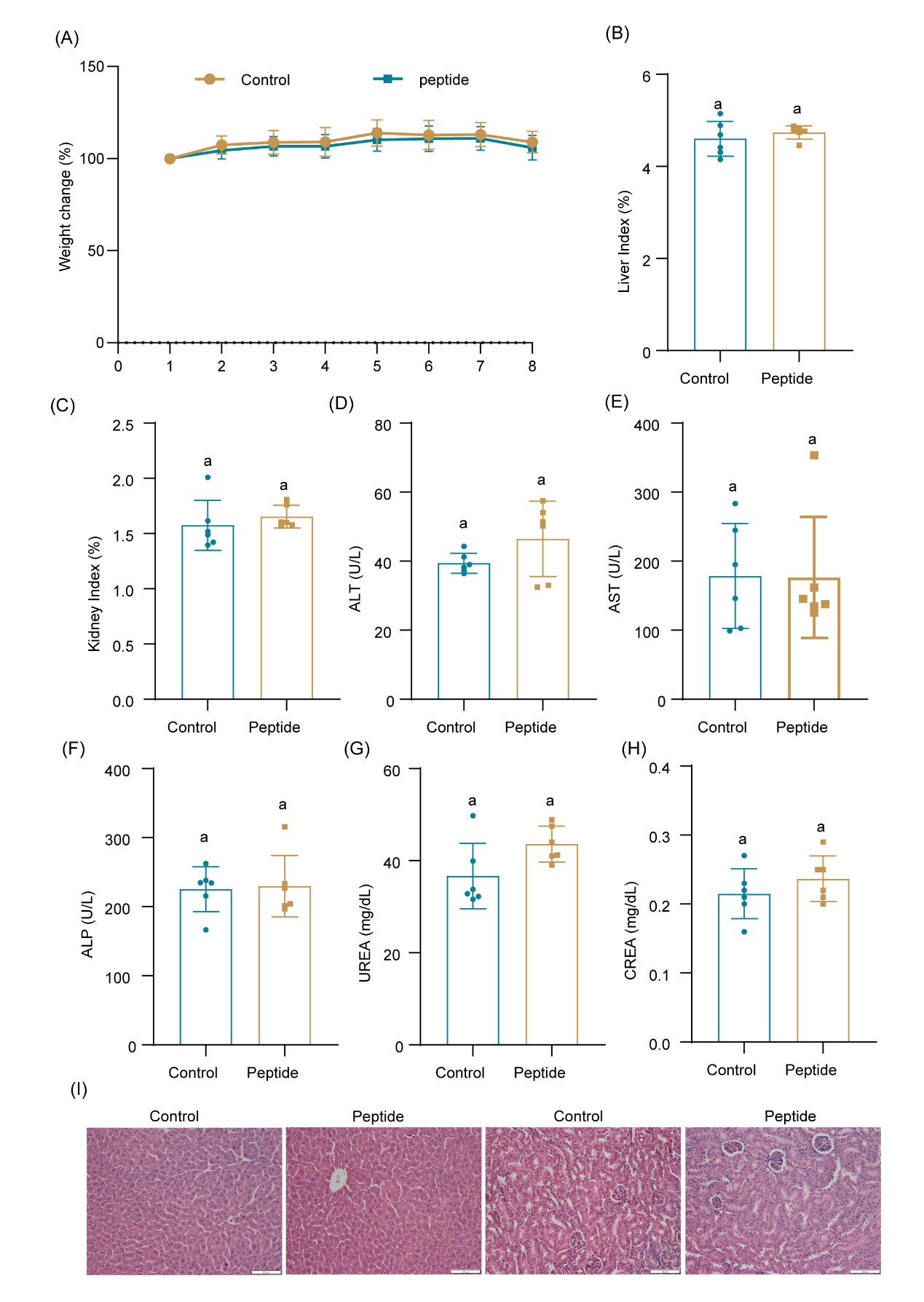


**Figure S8.** The biosafety was evaluated in mice by tail vein injection. (A) Body weight changes of BALB/C mice treated with different doses of SFK2. The mean initial body weight was used as a percentage. Data are expressed as mean ± standard deviation, n = 6. (B-C) Changes in relative organ weights of (b) liver and (C) kidney in mice after tail vein injection of SFK2 for 7 consecutive days. (D-H) Changes of liver and kidney related parameters in mice after tail vein injection of SFK2 for 7 consecutive days. (D-H) Data are expressed as mean ± standard deviation, n = 6. (I) Histomorphological observation of the liver and kidney of mice after 7 consecutive days of intraperitoneal injection of different concentrations of SFK2. Scale bar : 100 μm.


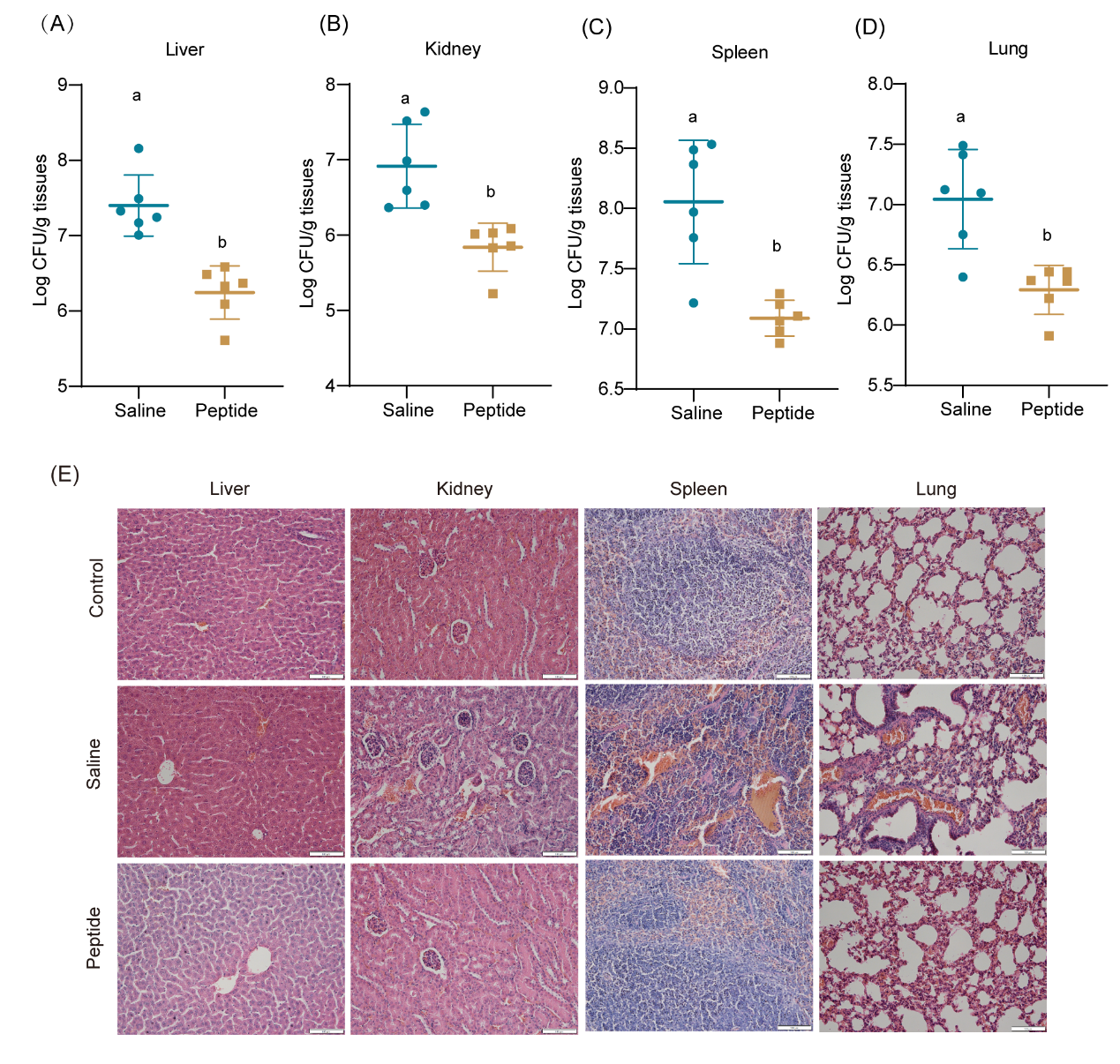


**Figure S9.** The therapeutic effect was evaluated in mice by tail vein injection. (A-D) The bacteria load in liver, kidney, spleen, and lung of mice treated with saline and SFK2. Data are expressed as mean ± standard deviation, n = 6. (E) Histopathological sections of liver, kidney, spleen, and lung of mice in healthy group, saline group, and peptide treatment group. Scale bar: 100 μm.
